# Immune Checkpoint Inhibitors and Immune-Related Adverse Events in Patients With Advanced Melanoma: A Systematic Review and Network Meta-analysis

**DOI:** 10.1001/jamanetworkopen.2020.1611

**Published:** 2020-03-25

**Authors:** Ching-Yuan Chang, Haesuk Park, Daniel C. Malone, Ching-Yu Wang, Debbie L. Wilson, Yu-Min Yeh, Sascha Van Boemmel-Wegmann, Wei-Hsuan Lo-Ciganic

**Affiliations:** 1 Department of Pharmaceutical Outcomes and Policy, University of Florida College of Pharmacy, Gainesville; 2 Center for Drug Evaluation and Safety, University of Florida College of Pharmacy, Gainesville; 3 Department of Pharmacotherapy, The University of Utah College of Pharmacy, Salt Lake City; 4 National Cheng-Kung University Hospital, Department of Internal Medicine, National Cheng Kung University College of Medicine, Tainan, Taiwan

## Abstract

**Importance:**

Since 2011, immune checkpoint inhibitors (ICIs) have been effective treatment options for advanced melanoma. Little is known about how risks of immune-related adverse events (irAEs) vary by ICIs.

**Objective:**

To compare the risk of irAEs across different treatment regimens for advanced melanoma using network meta-analysis.

**Data Sources:**

PubMed/MEDLINE, Embase, Web of Science, and Scopus were searched for all randomized clinical trial (RCT) articles published from January 1, 2010, through June 30, 2019.

**Study Selection:**

Studies included phases 2 and 3 RCTs in the treatment of advanced melanoma that compared ICIs (ipilimumab, nivolumab, and pembrolizumab) with chemotherapy drugs (eg, dacarbazine, carboplatin, and paclitaxel) or different ICI regimens.

**Data Extraction and Synthesis:**

Different treatment regimens were compared using bayesian network meta-analysis with Markov chain Monte Carlo simulation with noninformative prior distribution and random-effects generalized linear models.

**Main Outcomes and Measures:**

Primary outcomes were the cumulative incidence of any irAEs (regardless of severity) and severe irAEs (grades 3-5). Based on the pooled odds ratios (ORs) and 95% credible intervals (95% CrI), the probability of being associated with the lowest irAE risks was estimated for each treatment regimen.

**Results:**

Nine RCTs with 8 different treatment regimens for advanced melanoma and involving a total of 5051 patients were included. Overall, the 3 ICI treatment regimens associated with the lowest risk of any or severe irAEs were pembrolizumab, 2 mg/kg, every 3 weeks; nivolumab, 3 mg/kg, every 2 weeks; and pembrolizumab, 10 mg/kg, every 3 weeks. Compared with ipilimumab, 10 mg/kg, every 3 weeks, only nivolumab, 3 mg/kg, every 2 weeks, was associated with a decreased risk for any irAEs (OR, 0.34; 95% CrI, 0.13-0.94). A decreased risk for severe irAEs was observed for ipilimumab, 3 mg/kg, every 3 weeks (OR, 0.35; 95% CrI, 0.14-0.74); pembrolizumab, 10 mg/kg, every 2 weeks (OR, 0.22; 95% CrI, 0.05-0.95) and 10 mg/kg every 3 weeks (OR, 0.20; 95% CrI, 0.06-0.68); and nivolumab, 3 mg/kg, every 2 weeks (OR, 0.20; 95% CrI, 0.07-0.48) compared with ipilimumab, 10 mg/kg, every 3 weeks. An increased risk for severe irAEs was associated with nivolumab, 1 mg/kg, every 3 weeks combined with ipilimumab, 3 mg/kg, every 3 weeks compared with other ICI regimens (ORs ranging from 4.09 [95% CrI, 1.73-10.99] to 7.40 [95% CrI, 1.12-49.29]) except ipilimumab, 10 mg/kg, every 3 weeks.

**Conclusions and Relevance:**

These findings suggest that for patients with advanced melanoma at high risk of irAEs, pembrolizumab, 2 mg/kg, every 3 weeks, nivolumab, 3 mg/kg, every 2 weeks, and pembrolizumab, 10 mg/kg, every 3 weeks may be the preferred treatment regimens (with respect to irAE risks) among the ICI regimens reported, whereas ipilimumab, 10 mg/kg, every 3 weeks alone and nivolumab, 1 mg/kg, every 3 weeks combined with ipilimumab, 3 mg/kg, every 3 weeks should be used with caution. A network analysis may be valuable for clinical decision-making when evidence from head-to-head comparisons is lacking.

## Key Points

**Question:**

Does the risk of immune-related adverse events differ across immune checkpoint inhibitors used by patients with advanced melanoma?

**Findings:**

In this systematic review and network meta-analysis of randomized clinical trials of advanced melanoma treatment, the risk of immune-related adverse events varied by the immune checkpoint inhibitors used and by the different doses of these same immune checkpoint inhibitors. Pembrolizumab, 2 mg/kg, every 3 weeks and 10 mg/kg every 3 weeks as well as nivolumab, 3 mg/kg, every 2 weeks were the 3 immune checkpoint inhibitor regimens associated with the lowest risk of any or severe immune-related adverse events.

**Meaning:**

Results of this study suggest that network analysis comparing different treatment regimens for advanced melanoma may be valuable for clinical decision-making in the absence of evidence from randomized clinical trials with head-to-head comparisons.

## Teaser

This systematic review and meta-analysis evaluates the immune checkpoint inhibitor regimens used along with the incidence of adverse events reported in randomized clinical trials for treatment of advanced melanoma.

## Introduction

Melanoma, one of the serious forms of skin cancer, is the fifth most common cancer in the United States.^1^ In 2018, more than 91 000 individuals were newly diagnosed with melanoma.^2^ Most early-stage melanomas (stages I and II) are curable, with a 5-year survival rate of 98.4%.^1^ However, advanced melanoma (stages III and IV) with dacarbazine chemotherapy had a low 3-year survival rate of 12.2%.^3^ In recent years, immunotherapy with novel immune checkpoint inhibitors (ICIs) has revolutionized treatment approaches for advanced melanoma. Ipilimumab, the first ICI for treatment of advanced melanoma, was introduced to the US market in 2011. Since then, 2 additional ICIs (pembrolizumab and nivolumab) have been approved by the US Food and Drug Administration as a first-line treatment for advanced melanoma.

Randomized clinical trials (RCTs) have demonstrated superior survival rates associated with the ICIs compared with the historically used chemotherapy drugs among patients with advanced melanoma (eg, 3-year survival rate: 20.8% vs 12.2%).^3,4,5,6^ Despite the improved survival benefit associated with ICIs, concerns of immune-related adverse events (irAEs) associated with ICI regimens are growing because of their pharmacological mechanisms.^7^ By blocking the pathways that regulate the immune system, ICIs can increase the immune system’s activity, causing organ inflammation and thus increasing the risk of irAEs.^8^ ICI-associated irAEs can potentially involve multiple organs or systems, including the skin (eg, rash, pruritus), gastrointestinal tract (eg, diarrhea, colitis), endocrine (eg, hypothyroidism, hypophysitis), liver (eg, hepatitis), and lung (eg, pneumonitis).^3,4,5,6,9,10,11,12,13,14^ In the published RCTs, the occurrence of any irAEs varied from 54% to 96% among patients with advanced melanoma receiving ICIs.^4,5,6,9,10,11,12,13,14^ Without proper management, irAEs can be severe and life-threatening and may result in treatment discontinuation or failure.

Previous studies, including 1 network meta-analysis and 6 traditional meta-analyses, have examined the risks of irAEs associated with ICI therapy, but these studies mainly focused on patients with all types of cancer.^15,16,17,18,19,20,21^ In addition, these studies did not explicitly examine the risk of irAEs across different ICI regimens, which may vary by type of cancer. Little is known about how the risk of irAEs differs across ICI regimens among patients with advanced melanoma. Given the lack of head-to-head RCTs that directly compare different ICI regimens for advanced melanoma, we conducted a network meta-analysis. We combined direct and indirect evidence for pairwise comparisons of ICI regimens to assess the risks of irAEs in patients with advanced melanoma.

## Methods

### Data Sources and Searches

We followed the Preferred Reporting Items for Systematic Reviews and Meta-Analyses (PRISMA) guideline (eFigure 1 in the Supplement).^22^ Given that the first RCT using ICI for advanced melanoma was published in 2010, we systematically searched PubMed/MEDLINE, Embase, Web of Science, and Scopus for all RCT articles published from January 1, 2010, through June 30, 2019. The search strategy used a combination of terms, including controlled vocabulary (eg, MeSH [Medical Subject Headings] and Emtree) and text words developed in consultation with a pharmacy liaison research librarian (eTable 1 in the Supplement).

### Study Selection and Data Extraction

We identified RCTs (phases 2 and 3) that compared an ICI (ipilimumab, pembrolizumab, or nivolumab) or a combination therapy of 2 ICIs with (1) a chemotherapy drug (dacarbazine, carboplatin, or paclitaxel), (2) a different ICI, or (3) different doses of 1 of the ICIs for patients with advanced melanoma. All of the ICI treatment regimens reported in the RCTs were included to capture the potential treatment regimens used in clinical practice. *Advanced melanoma* was defined as stage III or IV melanoma or as surgically unresectable, with the cancer cells spread from the epidermis to the dermis of the skin or to distant organ sites.^23^

We excluded non–English-language articles, case reports, reviews, meta-analyses, editorials, commentary letters, conference proceedings, and extension analyses of previously published RCTs (eFigure 1 in the Supplement). After a comprehensive literature search and removal of duplicates, we (C.Y.C., C.Y.W.) independently reviewed the titles and abstracts of the articles and further screened their full text against our inclusion and exclusion eligibility criteria. We extracted the study information and details using a standardized spreadsheet (Microsoft Excel; Microsoft Corp). We resolved disagreements during the study selection and data extraction processes by consensus and consultation with a third reviewer (W.H.L.C.).

We extracted the following information for each eligible article: (1) study details (ie, year of publication, author names, country, and follow-up duration), (2) baseline characteristics of participants (eg, sex, age), and (3) interventions and outcomes (ie, population size of each group, treatment dose, and type and number of irAEs).

### Quality and Risk-of-Bias Assessment

Two of us (C.Y.C., C.Y.W.) independently evaluated the included studies for potential biases (eg, selection and performance bias) using the Cochrane Collaboration’s risk-of-bias assessment tool.^24^ Bias assessment was generated by Review Manager 5.3 (Cochrane Collaboration). We assessed publication bias using asymmetric distributions from the funnel plots when 4 or more studies for each comparison were available. Asymmetry of funnel plots visually indicates potential publication bias.^25^

### Outcome Measures

The primary outcomes included the cumulative incidence of any irAEs (severity grades 1-5) and severe irAEs (severity grades 3-5).^7^ Grades 1 and 2 referred to mild-to-moderate AEs, grade 3 indicated severe but not immediately life-threatening AEs, grade 4 indicated life-threatening AEs, and grade 5 indicated death-related events.^26^ We defined irAEs according to how each RCT reported its treatment-related AEs or irAEs. Treatment-related AEs and irAEs were highly associated with the trial’s intervention treatment.

The secondary outcomes included organ-specific irAEs and outcomes by AE severity. Dermatologic irAEs included pruritus, rash, and vitiligo. Gastrointestinal irAEs included diarrhea and colitis. Endocrine irAEs included hypothyroidism and hypophysis functioning. Hepatic irAEs included increased alanine aminotransferase level, increased aspartate aminotransferase level, and hepatitis. Pulmonary irAEs included pneumonitis.

### Data Synthesis and Statistical Analysis

We generated network plots depicting head-to-head comparisons between different treatment regimens using Stata, version 13.0 (StataCorp LLC). In a bayesian network meta-analysis, we simultaneously compared all treatment regimens for advanced melanoma using a Markov chain Monte Carlo simulation technique in WinBUGS, version 1.4 statistical software (MRC Biostatistics Unit). We applied noninformative previous distribution and random-effects generalized linear models with a logit link function, running 10 000 iterations in each of the 4 chains. We used pooled odds ratios (ORs) with 95% credible intervals (CrIs, the bayesian equivalent of CIs) to estimate the risk of irAEs across different treatment regimens. We generated forest plots using R, version 3.5.1 (R Project for Statistical Computing).

For the primary outcomes, we ranked the probability of a treatment regimen to be the best (ie, to be associated with the lowest risk of irAEs) by estimating the median (95% CrI) of the posterior distribution for the rank of each treatment regimen. The treatment ranked first was the best treatment regimen (ie, associated with the lowest risk of irAE). In each Markov chain Monte Carlo cycle, every treatment regimen was ranked according to the estimated OR. The probability of a certain treatment ranking as the best among all treatment regimens was estimated from the proportion of the cycles in which a given regimen was rated as first of the total Markov cycles.^27^

For the secondary outcomes, we conducted pairwise comparisons of ICI regimens to compare the associated risks or likelihood of irAEs. A statistically significant (*P* ≤ .05; 2-sided) OR of less than 1 suggested a lower associated risk for the reference treatment regimen compared with the comparison regimen, whereas a significant OR of greater than 1 suggested a greater associated risk for the reference regimen compared with the comparison regimen.

To ensure the robustness of the findings, we conducted a sensitivity analysis using the node-splitting method to evaluate the consistency between direct and indirect evidence to test for any violation of assumptions in the network analysis.^28,29^ Direct evidence was the effect estimated directly from 1 head-to-head RCT. Indirect evidence was the effect derived from the comparison of 2 separate head-to-head RCTs. We obtained posterior means (SDs) of the log ORs from direct and indirect evidence for the treatment comparison, and then we compared the inconsistency between direct and indirect effects (direct effect minus indirect effect) for each treatment comparison in R, version 3.5.1 (package: R2WinBUGS), and WinBUGS 1.4.^28^ If the *P* value measuring the agreement between direct and indirect evidence for each treatment comparison was significant (*P* < .05; 2-sided), then inconsistency was present between direct and indirect evidence, which indicated a violation of the network analysis assumption.

## Results

### Study and Patient Characteristics

The initial literature search yielded 4458 records, of which 2497 (56.0%) remained after removal of the duplicates. Screening of the titles and abstracts resulted in the exclusion of articles that were not RCTs (n = 1240); did not compare an ICI with a chemotherapy drug, another ICI, or a different dose of the same ICI regimen (n = 114); did not include patients with advanced melanoma (n = 1007); did not include an outcome of interest (n = 109); or were extension analyses of the included RCTs (n = 18). The network meta-analyses included 9 RCTs with 8 different treatment regimens for advanced melanoma^4,5,6,9,10,11,12,13,14^ (Figure; eFigure 1 in the Supplement).

**Figure.**
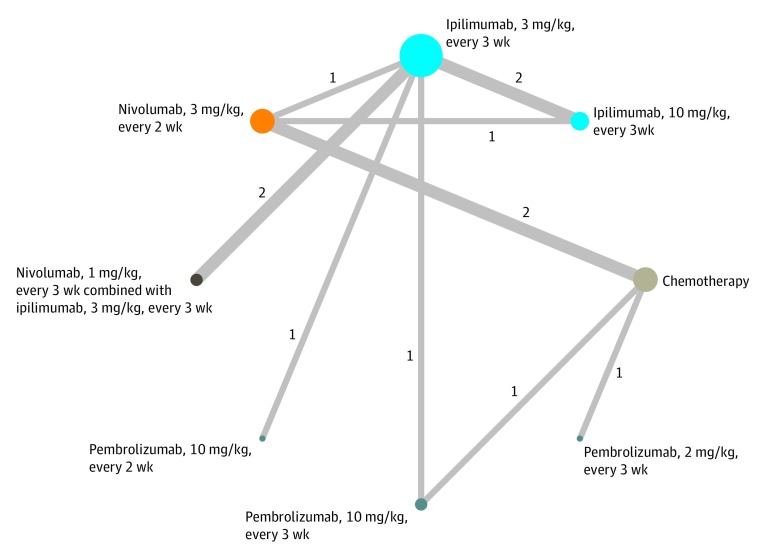
Network Diagram of 8 Treatment Regimens for Advanced Melanoma in 9 Trials Circular nodes indicate treatment regimens. The size of each circle corresponds with the number of participants, whereas the colors represent the types of treatment options. The width of the lines and the numbers next to these lines indicate the number of studies.

As shown in eTable 2 and eTable 3 in the Supplement, 8 RCTs were multinational studies,^4,5,6,9,10,12,13,14^ and 1 did not report the country of the study.^11^ Among 21 treatment regimens, 18 (85.7%) were ICIs (ie, ipilimumab, nivolumab, and pembrolizumab) with a median follow-up duration ranging from 5 to 38 months, and 3 (14.3%) were chemotherapy drugs (ie, carboplatin, dacarbazine, and/or paclitaxel) with a median follow-up duration of 5 to 28 months. Of the total 5051 participants, 2046 (40.5%) were women. The median patient age ranged from 55 to 67 years for those treated with ICI regimens (n = 4513) and from 62 to 66 years for those treated with chemotherapy drugs (n = 538).

### Risk-of-Bias Assessment and Publication Bias

The overall risk of bias was low for the 9 included RCTs^4,5,6,9,10,11,12,13,14^ (eFigure 2 in the Supplement). Four RCTs (44.4%) had high or unclear risk of performance and detection bias because of the lack of blinding of participants, personnel, and/or outcome assessment.^4,6,11,13^ Publication bias could not be assessed for any of the outcomes of interest because fewer than 4 studies were available for each treatment comparison.

### Primary Outcomes: Ranking the Probability of Treatment Regimen to Be the Best 

Overall, nivolumab, 3 mg/kg, by intravenous (IV) infusion every 2 weeks, pembrolizumab, 2 mg/kg, by IV infusion every 3 weeks, and pembrolizumab, 10 mg/kg, by IV infusion every 3 weeks were the 3 ICI treatment regimens most likely to rank as the best owing to being associated with the lowest risk of any or severe irAEs (Table 1; eTable 4 in the Supplement). Compared with 10 mg/kg of ipilimumab by IV infusion every 3 weeks, only 3 mg/kg of nivolumab every 2 weeks (OR, 0.34; 95% CrI, 0.13-0.94) was associated with a decreased risk of any irAEs (Table 2). In terms of the risk of severe irAEs (Table 3), compared with 10 mg/kg of ipilimumab every 3 weeks, a decreased risk was associated with ipilimumab, 3 mg/kg, by IV infusion every 3 weeks (OR, 0.35; 95% CrI, 0.14-0.74) followed by pembrolizumab, 10 mg/kg, by IV infusion every 2 weeks (OR, 0.22; 95% CrI, 0.05-0.95), nivolumab, 3 mg/kg, every 2 weeks (OR, 0.20; 95% CrI, 0.07-0.48), and pembrolizumab, 10 mg/kg, every 3 weeks (OR, 0.20; 95% CrI, 0.06-0.68).

**Table 1.** Ranking of the Probability of Being the Best Treatment Regimen^a^

Treatment regimen	Median rank (95% CrI)^b^
Pembrolizumab, 2 mg/kg, every 3 wk	1 (1-7)
Nivolumab, 3 mg/kg, every 2 wk	2 (1-6)
Pembrolizumab, 10 mg/kg, every 3 wk	3 (1-7)
Ipilimumab, 3 mg/kg, every 3 wk	4 (1-6)
Chemotherapy^c^	5 (2-7)
Pembrolizumab, 10 mg/kg, every 2 wk	6 (1-8)
Nivolumab, 1 mg/kg, every 3 wk and ipilimumab, 3 mg/kg, every 3 wk	7 (2-8)
Ipilimumab, 10 mg/kg, every 3 wk	7 (4-8)

Abbreviation: CrI, credible interval.

^a^ We ranked this probability by estimating the median (95% CrIs) of the posterior distribution for the rank of each treatment regimen. Some median ranks across different treatment regimens were the same because rank was an integer. The best treatment regimen was the one with the lowest risk of any immune-related adverse event.

^b^ Median rank refers to the median and the 95% CrI refers to the 95% CrI of the posterior distribution for the rank of each treatment regimen.

^c^ Immune-related adverse events were the outcomes associated with immune checkpoint inhibitors, not chemotherapy drugs (ie, carboplatin, dacarbazine, and paclitaxel). For patients receiving chemotherapy, the adverse events identified were associated with chemotherapy.

**Table 2.** Risk of Any Immune-Related Adverse Events Associated With Each Treatment Regimen^a^

Treatment regimen	Odds ratio (95% CrI)							
	Chemotherapy^b^	Ipilimumab		Pembrolizumab, 10 mg/kg, every 2wk	Nivolumab, 1 mg/kg, every 3 wk + ipilimumab 3 mg/kg every 3 wk	Nivolumab, 3 mg/kg, every 2 wk	Pembrolizumab	
		3 mg/kg every 3wk	10 mg/kg every 3wk				10 mg/kg every 3 wk	2 mg/kg every 3wk
Chemotherapy^b^	NA	1.13 (0.37-3.26)	0.50 (0.15-1.72)	0.79 (0.14-4.19)	0.79 (0.14-4.19)	1.44 (0.63-3.4)	1.21 (0.41-3.49)	1.74 (0.48-6.67)
Ipilimumab								
3 mg/kg every 3 wk	0.88 (0.30-2.70)	NA	0.44 (0.2-1.04)	0.71 (0.19-2.59)	0.71 (0.19-2.59)	1.29 (0.54-3.27)	1.08 (0.38-3.14)	1.55 (0.31-8.7)
10 mg/kg every 3wk	2.00 (0.58-6.45)	2.27 (0.94-4.96)	NA	1.60 (0.33-7.09)	1.60 (0.33-7.09)	2.91 (1.09-7.55)^c^	2.44 (0.68-8.36)	3.51 (0.62-20.48)
Pembrolizumab, 10 mg/kg, every 2 wk	1.25 (0.23-6.81)	1.42 (0.37-5.31)	0.62 (0.14-3.07)	NA	0.60 (0.13-3.67)	1.83 (0.39-9.23)	1.52 (0.29-7.99)	2.19 (0.28-18.94)
Nivolumab								
1 mg/kg every 3 wk and ipilimumab, 3 mg/kg, every 3 wk	2.05 (0.38-8.26)	2.31 (0.70-6.04)	1.02 (0.25-3.61)	1.66 (0.26-7.75)	NA	3.05 (0.7-10.59)	2.55 (0.51-9.88)	3.67 (0.46-23.96)
3 mg/kg every 2 wk	0.69 (0.29-1.60)	0.78 (0.30-1.90)	0.34 (0.13-0.94)^c^	0.55 (0.11-2.67)	0.33 (0.09-1.57)	NA	0.84 (0.27-2.55)	1.20 (0.27-5.83)
Pembrolizumab								
10 mg/kg every 3 wk	0.82 (0.28-2.44)	0.93 (0.32-2.67)	0.41 (0.12-1.50)	0.66 (0.12-3.56)	0.40 (0.10-2.08)	1.19 (0.38-3.81)	NA	1.44 (0.29-7.89)
2 mg/kg every 3 wk	0.58 (0.14-2.16)	0.65 (0.11-3.43)	0.29 (0.05-1.70)	0.46 (0.05-3.84)	0.28 (0.04-2.30)	0.84 (0.17-3.88)	0.70 (0.13-3.79)	NA

Abbreviations: CrI, credible interval; NA, not applicable.

^a^ The pooled odds ratios (95% CrIs) were the result of comparing the left-column treatment regimens with the top-row treatment regimens (the reference group).

^b^ Immune-related adverse events were the outcomes associated with immune checkpoint inhibitors, not chemotherapy drugs (ie, carboplatin, dacarbazine, and paclitaxel). For chemotherapy users, the adverse events identified were associated with chemotherapy. The pooled odds ratios were estimated from all direct and indirect comparisons.

^c^ Statistically significant.

**Table 3.** Risk of Severe Immune-Related Adverse Events Associated With Each Treatment Regimen^a^

Treatment regimen	Odds ratio (95% CrI)							
	Chemotherapy^b^	Ipilimumab		Pembrolizumab, 10 mg/kg, every 2 wk	Nivolumab, 1 mg/kg, every 3wk + ipilimumab, 3 mg/kg, every 3 wk	Nivolumab, 3 mg/kg, every 2 wk	Pembrolizumab	
		3 mg/kg every 3 wk	10 mg/kg every 3 wk				10 mg/kg every 3 wk	2 mg/kg every 3 wk
Chemotherapy^b^	NA	1.34 (0.48-4.04)	0.47 (0.14-1.50)	2.12 (0.43-11.28)	0.33 (0.08-1.30)	2.35 (1.04-5.56)^c^	2.31 (0.82-6.84)	2.42 (0.65-8.57)
Ipilimumab								
3 mg/kg every 3 wk	0.75 (0.25-2.08)	NA	0.35 (0.14-0.74)^c^	1.57 (0.45-5.71)	0.24 (0.09-0.58)^c^	1.75 (0.73-4.13)	1.72 (0.59-4.87)	1.82 (0.33-9.13)
10 mg/kg every 3 wk	2.13 (0.67-7.13)	2.86 (1.35-6.95)^c^	NA	4.48 (1.06-22.02)^c^	0.70 (0.21-2.41)	4.96 (2.09-13.53)^c^	4.90 (1.48-18.14)^c^	5.21 (0.93-28.90)
Pembrolizumab, 10 mg/kg, every 2 wk	0.47 (0.09-2.31)	0.64 (0.18-2.24)	0.22 (0.05-0.95)^c^	NA	0.16 (0.03-0.70)^c^	1.12 (0.24-5.06)	1.10 (0.21-5.48)	1.15 (0.14-8.61)
Nivolumab								
1 mg/kg every 3 wk and ipilimumab, 3 mg/kg, every 3 wk	3.03 (0.77-12.79)	4.09 (1.73-10.99)^c^	1.43 (0.42-4.87)	6.37 (1.43-34.00)^c^	NA	7.12 (2.19-26.51)^c^	6.97 (1.82-30.15)^c^	7.40 (1.12-49.29)^c^
3 mg/kg every 2 wk	0.43 (0.18-0.96)^c^	0.57 (0.24-1.37)	0.20 (0.07-0.48)^c^	0.90 (0.20-4.24)	0.14 (0.04-0.46)^c^	NA	0.98 (0.32-3.01)	1.04 (0.21-4.54)
Pembrolizumab								
10 mg/kg every 3 wk	0.43 (0.15-1.22)	0.58 (0.21-1.69)	0.20 (0.06-0.68)^c^	0.91 (0.18-4.81)	0.14 (0.03-0.55)^c^	1.02 (0.33-3.16)	NA	1.05 (0.2-5.12)
2 mg/kg every 3 wk	0.41 (0.12-1.53)	0.55 (0.11-3.07)	0.19 (0.03-1.08)	0.87 (0.12-7.19)	0.14 (0.02-0.89)^c^	0.97 (0.22-4.66)	0.95 (0.20-5.04)	NA

Abbreviations: CrI, credible interval; NA, not applicable.

^a^ The pooled odds ratios (95% CrIs) were the result of comparing the left-column treatment regimens with the top-row treatment regimens (the reference group).

^b^ Immune-related adverse events were the outcomes associated with immune checkpoint inhibitors, not chemotherapy drugs (ie, carboplatin, dacarbazine, and paclitaxel). For chemotherapy users, the adverse events identified were associated with chemotherapy. The pooled odds ratios were estimated from all direct and indirect comparisons.

^c^ Statistically significant.

As shown in Table 3, compared with 1 mg/kg of nivolumab by IV infusion every 3 weeks combined with 3 mg/kg of ipilimumab every 3 weeks, a decreased risk of severe irAEs was associated with ipilimumab, 3 mg/kg, every 3 weeks (OR, 0.24; 95% CrI, 0.09-0.58), pembrolizumab, 10 mg/kg, every 2 weeks (OR, 0.16; 95% CrI, 0.03-0.70), nivolumab, 3 mg/kg, every 2 weeks (OR, 0.14; 95% CrI, 0.04-0.46), pembrolizumab, 10 mg/kg, every 3 weeks (OR, 0.14; 95% CrI, 0.03-0.55), and pembrolizumab, 2 mg/kg, every 3 weeks (OR, 0.14; 95% CrI, 0.02-0.89). Of the ICI regimens, only nivolumab, 3 mg/kg, every 2 weeks (OR, 0.43; 95% CrI, 0.18-0.96) was associated with a lower risk of severe irAEs compared with chemotherapy drugs.

### Secondary Outcomes: Ranking the Probability of Treatment Regimen to Be the Best

The ranking probability of an ICI treatment regimen having the lowest risk varied by organ system and by AE severity. The statistically significant secondary outcomes are described herein.

For dermatologic irAEs, among the ICI regimens, nivolumab, 3 mg/kg, every 2 weeks had the lowest risk of any pruritus (median rank, 2; 95% CrI, 2-6) and any rash (median rank, 4; 95% CrI, 2-8) (Table 4) and was associated with the second lowest risk of severe pruritus and severe rash among the ICI regimens (median rank, 3; 95% CrI, 1-6) (eTable 5 and eTable 6 in the Supplement). In addition, an associated lower risk of severe rash (OR, 0.06; 95% CrI, 0.004-0.49) was observed for nivolumab, 3 mg/kg, every 2 weeks compared with the ICI combination of nivolumab, 1 mg/kg, every 3 weeks and ipilimumab, 3 mg/kg, every 3 weeks (eTable 6 in the Supplement). Except for pembrolizumab, 10 mg/kg, every 2 weeks, the ICI regimens were associated with a higher risk of any pruritus compared with chemotherapy drugs (range of all ICI ORs = 3.15-6.88) (eTable 6 in the Supplement).

**Table 4.** Ranking of the Probability of Being the Best Treatment Regimen, by System or Organ Immune-Related Adverse Events^a^

System or Organ Immune-Related Adverse Event	Treatment regimen, median rank (95% CrI)^b^							
	Chemotherapy^c^	Ipilimumab		Pembrolizumab, 10 mg/kg, every 2 wk	Nivolumab, 1 mg/kg, every 3 wk and ipilimumab, 3 mg/kg, every 3 wk	Nivolumab, 3 mg/kg, every 2 wk	Pembrolizumab	
		3 mg/kg every 3 wk	10 mg/kg every 3 wk				10 mg/kg every 3 wk	2 mg/kg every 3 wk
Dermatologic irAEs								
Pruritus	1 (1-2)	5 (3-8)	6 (2-8)	5 (1-8)	6 (2-8)	2 (2-6)	6 (2-8)	6 (2-8)
Rash	1 (1-3)	6 (3-7)	4 (2-8)	6 (1-8)	8 (2-8)	4 (2-8)	4 (2-8)	4 (1-8)
Vitiligo	1 (1-4)	3 (1-6)	3 (1-8)	7 (2-8)	4 (1-8)	5 (2-8)	7 (3-8)	6 (1-8)
Gastrointestinal irAEs								
Diarrhea	2 (1-5)	6 (4-7)	7 (5-8)	5 (1-7)	8 (6-8)	2 (1-5)	4 (1-6)	3 (1-7)
Colitis	1 (1-6)	6 (4-7)	5 (3-7)	3 (1-6)	7 (4-7)	2 (1-6)	4 (1-7)	NA^d^
Endocrine irAEs								
Hypothyroidism	1 (1-3)	2 (1-5)	3 (2-7)	7 (3-8)	5 (2-8)	4 (2-8)	7 (3-8)	7 (2-8)
Hypophysis	NA^d^	4 (3-6)	4 (2-6)	2 (1-5)	6 (3-6)	2 (1-4)	3 (1-6)	NA^d^
Liver irAEs								
Increased ALT level	1 (1-4)	2 (1-3)	4 (3-5)	NA^d^	5 (2-5)	3 (1-4)	NA^d^	NA^d^
Increased AST level	1 (1-4)	2 (1-3)	4 (3-5)	NA^d^	4 (2-5)	3 (1-4)	NA^d^	NA^d^
Hepatitis	NA^d^	2 (1-4)	4 (1-4)	2 (1-4)	NA^d^	NA^d^	3 (1-4)	NA^d^
Pulmonary irAEs								
Pneumonitis	1 (1-6)	3 (1-5)	7 (2-7)	3 (1-7)	5 (2-7)	3 (1-6)	6 (2-7)	NA^d^

Abbreviations: ALT, alanine aminotransferase; AST, aspartate aminotransferase; CrI, credible interval; irAE, immune-related adverse event; NA, not applicable.

^a^ We ranked this probability by estimating the median (95% CrIs) of the posterior distribution for the rank of each treatment regimen. Some median ranks across different treatment regimens were the same because rank was an integer. The best treatment regimen was the one with the lowest risk of any irAE.

^b^ Median rank refers to the median and the 95% CrI refers to the 95% CrI of the posterior distribution for the rank of each treatment regimen.

^c^ Immune-related AEs were the outcomes associated with immune checkpoint inhibitors, not chemotherapy drugs (ie, carboplatin, dacarbazine, and paclitaxel). For chemotherapy users, the adverse events identified were associated with chemotherapy.

^d^ No specific individual irAE was reported in that treatment regimen in the included studies, so the indirect evidence could not be generated. For example, pneumonitis was not reported in any included studies comparing pembrolizumab, 2 mg/kg, every 3 weeks with other treatments, so the indirect evidence of pneumonitis from pembrolizumab, 2 mg/kg, every 3 weeks could not be generated by linking that regimen with other treatments.

For gastrointestinal irAEs, the ICI regimen with the lowest risk of any diarrhea, any colitis (Table 4), and severe colitis (eTable 5 in the Supplement) was nivolumab, 3 mg/kg, every 2 weeks. In addition, nivolumab, 3 mg/kg, every 2 weeks was associated with the lowest risk of any diarrhea compared with ipilimumab, 3 mg/kg, every 3 weeks (OR, 0.48; 95% CrI, 0.30-0.78), ipilimumab, 10 mg/kg, every 3 weeks (OR, 0.39; 95% CrI, 0.23-0.61), and nivolumab, 1 mg/kg, every 3 weeks combined with ipilimumab, 3 mg/kg, every 3 weeks (OR, 0.30; 95% CrI, 0.15-0.58) (eTable 7 in the Supplement). Compared with chemotherapy drugs, nivolumab, 1 mg/kg, every 3 weeks combined with ipilimumab, 3 mg/kg, every 3 weeks (OR, 3.27; 95% CrI, 1.46-7.22) and the monotherapy drugs ipilimumab, 10 mg/kg, every 3 weeks (OR, 2.49; 95% CrI, 1.29-4.94) and ipilimumab, 3 mg/kg, every 3 weeks (OR, 2.02; 95% CrI, 1.05-3.71) (eTable 7 in the Supplement) were associated with a higher risk of any diarrhea.

For endocrine irAEs, ipilimumab, 3 mg/kg, every 3 weeks was associated with the lowest risk for any hypothyroidism (median rank, 2; 95% CrI, 1-5), and nivolumab, 3 mg/kg, every 2 weeks was associated with the lowest risk for any hypophysis (median rank, 2; 95% CrI, 1-4) (Table 4; eTable 8 in the Supplement). Moreover, of the ICI regimens, nivolumab, 3 mg/kg, every 2 weeks was associated with the lowest risk of severe hypothyroidism (median rank, 3; 95% CrI, 1-7) and severe hypophysis (median rank, 2; 95% CrI, 1-5) (eTable 5 in the Supplement).

For hepatic irAEs, ipilimumab, 3 mg/kg, every 3 weeks was associated with the lowest risk among the ICI regimens (Table 4; eTable 5 in the Supplement). Compared with ipilimumab, 3 mg/kg, every 3 weeks, ipilimumab, 10 mg/kg, every 3 weeks was associated with a higher risk of any increase in alanine aminotransferase level (OR, 4.79; 95% CrI, 1.02-28.17) and any increase in aspartate aminotransferase level (OR, 5.28; 95% CrI, 1.21-33.28). Compared with ipilimumab, 3 mg/kg, every 3 weeks, 1 mg/kg of nivolumab every 3 weeks combined with 3 mg/kg of ipilimumab every 3 weeks was associated with a higher risk of any increase (OR, 5.62; 95% CrI, 1.19-21.46) or severe increase (OR, 7.15; 95% CrI, 1.57-47.37) in alanine aminotransferase level and with any increase (OR, 5.12; 95% CrI, 1.21-19.11) or severe increase (OR, 10.42; 95% CrI, 1.72-92.95) in aspartate aminotransferase level (eTable 9 in the Supplement).

For pulmonary irAEs, 10 mg/kg of pembrolizumab every 2 weeks was associated with the lowest risk among the ICI regimens for any risk (median rank, 3; 95% CrI, 1-7) or severe risk (median rank, 2; 95% CrI, 1-7) of pneumonitis (Table 4; eTable 5 and eTable 10 in the Supplement).

### Sensitivity Analysis: Consistency Between Direct and Indirect Evidence

No significant inconsistency was observed between direct and indirect studies (eTable 11 and eTable 12 in the Supplement). However, the consistency analysis was not applicable when no direct head-to-head study evidence was available for a specific treatment regimen.

## Discussion

The network analysis yielded 3 important findings regarding ICI use and risk of irAEs among patients with advanced melanoma. First, the 3 ICI regimens associated with the lowest risk of (and thus with the best safety profiles for) any or severe irAEs were nivolumab, 3 mg/kg, every 2 weeks, pembrolizumab, 2 mg/kg, every 3 weeks, and pembrolizumab, 10 mg/kg, every 3 weeks. In contrast, ipilimumab, 10 mg/kg, every 3 weeks alone and nivolumab, 1 mg/kg, every 3 weeks combined with ipilimumab, 3 mg/kg, every 3 weeks were associated with higher risks compared with the other ICI regimens. Second, within the various organ systems and AE severities, different ICI regimens were ranked as being associated with the lowest risk of irAEs. Nivolumab, 3 mg/kg, every 2 weeks was associated with a lower risk of dermatologic, gastrointestinal, and endocrine irAEs (regardless of AE severity) compared with other ICI regimens. Ipilimumab, 3 mg/kg, every 3 weeks was associated with a lower risk of any or severe liver irAEs, and pembrolizumab, 10 mg/kg, every 2 weeks was associated with a lower risk of any or severe pulmonary irAEs. Third, ICI regimens were associated with a higher risk of any pruritus and diarrhea compared with traditional chemotherapy regimens.

These findings were generally consistent with the results from 3 previous studies in terms of nivolumab and pembrolizumab being the ICIs with the best safety profile for irAEs.^15,16,21^ In addition, chemotherapy had the third best safety profile for any AEs, although it was ranked as having the best or second-best safety profiles for AEs within the various organ systems and AE severities. The distribution of any AEs and organ-specific AEs across different treatment regimens may explain this discrepancy. More patients receiving chemotherapy than those receiving ICI therapy experienced a treatment-related AE within an organ system, but they typically only had 1 AE. In contrast, patients receiving ICI therapy who had an irAE often had more than 1 AE. Therefore, the number of patients receiving chemotherapy who experienced any AEs was larger than the number of those receiving ICI therapy who experienced any irAEs.

The present study thoroughly compared different ICI dose regimens and examined their irAE risk. Still, the results of this study differed from previous findings in several aspects. For instance, previous studies provided limited evidence on the variation of risks of different doses of pembrolizumab.^15,16,21^ In the present study, pembrolizumab, 2 mg/kg, every 3 weeks and 10 mg/kg every 3 weeks had better safety profiles for irAEs compared with pembrolizumab, 10 mg/kg, every 2 weeks. Moreover, a previous network analysis ranked chemotherapy drugs as being associated with a lower risk of irAEs compared with ipilimumab.^15^ We found ipilimumab, 3 mg/kg, every 3 weeks to be associated with a lower risk of any or severe irAEs than chemotherapy drugs, but we found ipilimumab, 10 mg/kg, every 3 weeks to be associated with a higher risk compared with chemotherapy drugs. The ipilimumab regimen of 10 mg/kg every 3 weeks was used less often for advanced melanoma in the current clinical practice.^30^

Past studies focused on examining individual irAEs by severity among patients with all types of cancer.^15,16,21^ Unlike studies^15^ that included ICIs that were not approved for melanoma, the present study ranked the treatment options approved by the Food and Drug Administration for melanoma only. This distinction is important because the rankings of the treatment options for advanced melanoma were different from those of other cancers in previous studies. For example, Xu et al^15^ showed that atezolizumab was the best ICI with the lowest risk of irAEs among patients with all types of cancer. However, atezolizumab was not approved by the Food and Drug Administration as a treatment option for advanced melanoma.

The presentations of irAEs are immune-mediated damages of normal tissue in various organ systems. Possible explanations for the differences in the risk of irAEs may be the different mechanisms of action of each medication and the combined use of ICIs.^31^ Ipilimumab is a cytotoxic T-lymphocyte–associated protein 4 inhibitor that enhances T-cell priming. Pembrolizumab and nivolumab are PD-1 (programmed cell death 1) inhibitors that reinvigorate preexisting T-cell responses.^32^ It is possible that irAEs might be more predominant through priming than through reinvigorating T-cell response. Moreover, it is common to observe more AEs when drugs with different mechanisms are combined. Illustrating this point in this study, nivolumab, 1 mg/kg, every 3 weeks combined with ipilimumab, 3 mg/kg, every 3 weeks had the highest risk of severe irAEs compared with the independent use of ipilimumab, pembrolizumab, or nivolumab.

Although ICIs have become the first-line treatment options for advanced melanoma in accordance with the current National Comprehensive Cancer Network guidelines,^33^ clinicians must tailor treatment regimens to maximize the treatment advantages while reducing any AEs associated with treatment discontinuation or failure. In a network meta-analysis that included 12 RCTs focusing on all types of cancers, the combination of 2 ICI regimens or the use of nivolumab or pembrolizumab alone compared with ipilimumab was associated with a statistically significant survival advantage.^34^ These advantages must be considered along with the risks. For instance, the present study found that the combination of 2 ICI regimens was associated with a higher risk of irAEs compared with other ICI regimens alone. These findings provide additional evidence and insights for better clinical practice guidance on ICI use in terms of irAEs, especially in patients at high risk.^35,36^ Specifically, for individuals with preexisting autoimmune diseases, initiating ICI regimens with a lower risk of any or severe irAEs is recommended to avoid irAEs. Also critical is regularly monitoring patients with advanced melanoma (eg, testing aspartate aminotransferase or alanine aminotransferase level every week) and then adjusting doses if needed.^7^

To our knowledge, this study was the first network meta-analysis that thoroughly examined the risk of irAEs by different ICI regimens for advanced melanoma. It also examined individual irAEs by organ and AE severity.

### Limitations

This study has several limitations. First, the RCTs used different terms to describe irAEs. We reviewed the grading system and terminology used in reporting irAEs and found them to be consistent and compatible. In addition, awareness and reporting of irAEs may have increased over time as the number of patients receiving ICI therapy increased. Second, the wide CrIs of the risk measures in this study could be attributed to the small number of studies, small sample sizes in some studies, and different methods and reporting standards for ICI-related AEs. We were not able to examine consistency between direct and indirect evidence using the node-splitting method when no direct head-to-head studies were available. Similarly, we were not able to examine publication bias because fewer than 4 studies for each treatment comparison were available. However, we included the findings from all of the registered RCTs evaluating ICI use in patients with advanced melanoma during the study period.

Third, 5 of the 9 RCTs did not report follow-up duration for each treatment regimen,^3,4,5,6,12^ so the results were reported as cumulative incidence. However, we did not observe a higher risk of any irAEs in the studies with longer follow-up durations. The findings were unlikely to be confounded by the follow-up duration. Fourth, this network analysis compared irAEs across ICI regimens. We did not compare individual ICIs with individual chemotherapy drugs because of the limited sample sizes. Fifth, our ability to report the ranking of a treatment regimen’s probability to have the lowest risk of each irAE was limited by the specific irAEs reported in the RCTs. Thus, we could not evaluate and rank some treatment options, and the CrIs for some treatment options were wide because of the small number of studies available for ranking.

## Conclusions

Nivolumab, 3 mg/kg, every 2 weeks as well as pembrolizumab, 2 mg/kg, every 3 weeks and 10 mg/kg every 3 weeks may be the preferred ICI regimens for advanced melanoma given that they are associated with a low risk of irAEs. This finding may be especially important for individuals with autoimmune diseases or those who use immunosuppressants. Ipilimumab, 10 mg/kg, every 3 weeks alone and nivolumab, 1 mg/kg, every 3 weeks combined with ipilimumab, 3 mg/kg, every 3 weeks should be used with caution and close monitoring. A network analysis of different treatment regimens may be valuable for clinical decision-making in the absence of evidence from RCTs.

## Notes

Supplement. **eTable 1.** Search Strategies **eTable 2.** Characteristics of Included Randomized Controlled Trials (n = 9) for Advanced Melanoma Events **eTable 3.** Frequencies of Any Immune-Related AEs in the Included Studies (n = 9) **eTable 4.** Probability Ranking of Being the Best Treatment Regimen With the Lowest Risk of Severe Immune-Related Adverse Events **eTable 5.** Probability Ranking of Being the Best Treatment Regimen With the Lowest Risk of Individual Severe Immune-Related Adverse Events **eTable 6.** Secondary Outcomes of Individual Skin-Related Immune-Related Adverse Events by Severity **eTable 7.** Secondary Outcomes of Individual Gastrointestinal Tract-Related Immune-Related Adverse Events by Severity **eTable 8.** Secondary Outcomes of Individual Endocrine-Related Immune-Related Adverse Events by Severity **eTable 9.** Secondary Outcomes of Individual Liver-Related Immune-Related Adverse Events by Severity **eTable 10**. Secondary Outcomes of Individual Pulmonary-Related Immune-Related Adverse Events by Severity **eTable 11.** Node-Splitting Analysis of the Primary Outcome: Any Immune-Related Adverse Events **eTable 12.** Node-Splitting Analysis of the Primary Outcome: Severe Immune-Related Adverse Events **eFigure 1.** Preferred Reporting Items for Systematic Reviews and Meta-Analyses (PRISMA) Flow Diagram: Literature Search, Study Identification, Selection, and Exclusion **eFigure 2.** Risk of Bias Assessment for the 9 Included Randomized Controlled Trials **eReferences.**
