# Supplementary material for: Immune Checkpoint Inhibitors and Immune-Related Adverse Events in Patients With Advanced Melanoma: A Systematic Review and Network Meta-analysis
Source: JAMA Netw Open. 2020 Mar 25;3(3):e201611. doi: 10.1001/jamanetworkopen.2020.1611 (PMC7097702; doi:10.1001/jamanetworkopen.2020.1611)
Supplement: Supplement. — eTable 1. Search Strategies eTable 2. Characteristics of Included Randomized Controlled Trials (n = 9) for Advanced Melanoma Events eTable 3. Frequencies of Any Immune-Related AEs in the Included Studies (n = 9) eTable 4. Probability Ranking of Being the Best Treatment Regimen With the Lowest Risk of Severe Immune-Related Adverse Events eTable 5. Probability Ranking of Being the Best Treatment Regimen With the Lowest Risk of Individual Severe Immune-Related Adverse Events eTable 6. Secondary Outcomes of Individual Skin-Related Immune-Related Adverse Events by Severity eTable 7. Secondary Outcomes of Individual Gastrointestinal Tract-Related Immune-Related Adverse Events by Severity eTable 8. Secondary Outcomes of Individual Endocrine-Related Immune-Related Adverse Events by Severity eTable 9. Secondary Outcomes of Individual Liver-Related Immune-Related Adverse Events by Severity eTable 10. Secondary Outcomes of Individual Pulmonary-Related Immune-Related Adverse Events by Severity eTable 11. Node-Splitting Analysis of the Primary Outcome: Any Immune-Related Adverse Events eTable 12. Node-Splitting Analysis of the Primary Outcome: Severe Immune-Related Adverse Events eFigure 1. Preferred Reporting Items for Systematic Reviews and Meta-Analyses (PRISMA) Flow Diagram: Literature Search, Study Identification, Selection, and Exclusion eFigure 2. Risk of Bias Assessment for the 9 Included Randomized Controlled Trials eReferences. [file jamanetwopen-3-e201611-s001.pdf]

# Supplementary Online Content

Chang C-Y, Park H, Malone DC, et al. Immune checkpoint inhibitors and immune-related adverse events in patients with advanced melanoma: a systematic review and network meta-analysis. *JAMA Netw Open.* 2020;3(3):e201611. doi:10.1001/jamanetworkopen.2020.1611

**eTable 1.** Search Strategies

**eTable 2.** Characteristics of Included Randomized Controlled Trials (n=9) for Advanced Melanoma Events

**eTable 3.** Frequencies of Any Immune-Related AEs in the Included Studies (n=9)

**eTable 4.** Probability Ranking of Being the Best Treatment Regimen With the Lowest Risk of Severe Immune-Related Adverse Events

**eTable 5.** Probability Ranking of Being the Best Treatment Regimen With the Lowest Risk of Individual Severe Immune-Related Adverse Events

**eTable 6.** Secondary Outcomes of Individual Skin-Related Immune-Related Adverse Events by Severity

**eTable 7.** Secondary Outcomes of Individual Gastrointestinal Tract-Related Immune-Related Adverse Events by Severity

**eTable 8.** Secondary Outcomes of Individual Endocrine-Related Immune-Related Adverse Events by Severity

**eTable 9.** Secondary Outcomes of Individual Liver-Related Immune-Related Adverse Events by Severity

**eTable 10.** Secondary Outcomes of Individual Pulmonary-Related Immune-Related Adverse Events by Severity

**eTable 11.** Node-Splitting Analysis of the Primary Outcome: Any Immune-Related Adverse Events

**eTable 12.** Node-Splitting Analysis of the Primary Outcome: Severe Immune-Related Adverse Events

**eFigure 1.** Preferred Reporting Items for Systematic Reviews and Meta-Analyses (PRISMA) Flow Diagram: Literature Search, Study Identification, Selection, and Exclusion

**eFigure 2.** Risk of Bias Assessment for the 9 Included Randomized Controlled Trials

**eReferences.**

This supplementary material has been provided by the authors to give readers additional information about their work.

**eTable 1.** Search Strategies

| Sources | Search strategies |
|---|---|
| PubMed/MEDLINE | ((("advanced melanoma" OR (("Melanoma"[Mesh] OR melanoma) AND ( metastatic OR unresectable OR advanced OR Metastasis OR metastasize*))))) AND (((("Ipilimumab"[Mesh] OR ipilimumab OR "Nivolumab"[Mesh] OR opdivo OR yervoy OR keytruda OR "pembrolizumab" [Supplementary Concept] OR Keytruda OR lambrolizumab OR "immune checkpoint inhibitor" OR "immune checkpoint inhibitors" OR CTLA-4 OR CTLA 4 Antigen OR "Programmed Cell Death 1 Receptor"[Mesh] OR PD-1 Receptor OR "CTLA-4 Antigen"[Mesh])))) |
| Elsevier EMBASE | ('melanoma metastasis'/exp OR 'melanoma metastasis' OR 'metastatic melanoma'/exp OR 'metastatic melanoma' OR 'advanced melanoma'/exp OR 'advanced melanoma' OR 'unresectable melanoma' OR 'metastasized melanoma') AND ('mdx 101' OR 'mdx010' OR 'mdx101' OR 'strentarga' OR 'yervoy' OR 'nivolumab'/exp OR 'bms 936558' OR 'bms936558' OR 'cmab 819' OR 'cmab819' OR 'mdx 1106' OR 'mdx1106' OR 'nivolumab' OR 'ono 4538' OR 'ono4538' OR 'opdivo' OR 'pembrolizumab'/exp OR 'keytruda' OR 'lambrolizumab' OR 'mk 3475' OR 'mk3475' OR 'pembrolizumab' OR 'sch 900475' OR 'sch900475' OR 'cytotoxic t lymphocyte antigen 4'/exp OR 'cd152 antigen' OR 'ctla 4' OR 'ctla 4 antigen' OR 'ctla-4 antigen' OR 'antigen cd152' OR 'ctla4' OR 'cytotoxic t lymphocyte antigen 4' OR 'cytotoxic t lymphocyte associated antigen 4' OR 'programmed death 1 receptor'/exp OR 'cd279 antigen' OR 'pd 1 protein' OR 'pdcd1 protein' OR 'antigen cd279' OR 'programmed cell death 1 protein' OR 'programmed cell death 1 receptor' OR 'programmed cell death protein 1' OR 'programmed death 1 protein' OR 'programmed death 1 receptor' OR 'programmed death protein 1' OR 'protein pd 1' OR 'protein pdcd1' OR 'protein programmed cell death 1' OR 'protein programmed death 1' OR 'immune checkpoint inhibitors') |
| Thomson Reuters Web of Science | ((TS=("Advanced melanoma" OR "Metastatic melanoma" OR "Unresectable melanoma"))) AND ((TS=(ipilimumab OR pembrolizumab OR nivolumab or Yervoy or keytruda or opdivo or “Immune checkpoint inhibitors” ))) AND (TS=(controlled trial) OR TS=(prospective stud) OR TI=(random) OR TS=(placebo) OR TS=(double blind) OR TS=(comparative stud)) |
| Elsevier Scopus | ( TITLE-ABS-KEY ( "advanced melanoma" OR "Metastatic melanoma" OR "Unresectable melanoma" ) ) AND (TITLE-ABS-KEY ( ipilimumab OR nivolumab OR pembrolizumab ) ) AND (TITLE-ABS-KEY ( "clinical trials" OR "clinical trials as a topic" OR "randomized controlled trial" OR "Randomized Controlled Trials as Topic" OR "random allocation" OR "Double-Blind Method" OR "Cross-Over Studies" OR "Placebos" OR "multicenter study" OR "double blind procedure" OR "crossover procedure" OR "clinical trial" OR "controlled study" OR "randomization" OR "placebo" ) ) |

**eTable 2.** Characteristics of Included Randomized Controlled Trials (n=9) for Advanced Melanoma Events

| Author, year | Country | Intervention (I) (dosage) | Comparator (C) (dosage) | Follow-up months | Patients, n | | Age, Median | | Female, % | |
|---|---|---|---|---|---|---|---|---|---|---|
| | | | | | I | C | I | C | I | C |
| Wolchok, 2010[1] | Multiple | IPI (3 mg/kg/q3 wks) | IPI (10 mg/kg/q3 wks) | 8.7(I)/10.7(C) | 72 | 72 | 59 | 56 | 33 | 39 |
| Robert, 2014[2] | Multiple | NIV (3 mg/kg/q2 wks) | DTIC | 5.2-16.7 | 210 | 208 | 64 | 66 | 42 | 40 |
| Robert, 2015[3] | NR | PEM (10 mg/kg/q2 wks) | IPI (3 mg/kg/q3 wks) | 7.9 | 279 | 278 | 61 | 62 | 42 | 42 |
| | | PEM (10 mg/kg/q3 wks) | | | 277 | 278 | 63 | 62 | 37 | 42 |
| Weber, 2015[4] | Multiple | NIV (3 mg/kg/q2 wks) | DTIC or PTX + CBP | 24 | 272 | 133 | 62 | 62 | 35 | 36 |
| Hodi, 2017[5] | Multiple | NIV (1 mg/kg/q3 wks) + IPI (3 mg/kg/q3 wks) | IPI (3 mg/kg/q3 wks) | 24.0(I)/23.0(C) | 95 | 47 | 64 | 67 | 34 | 32 |
| Ascierto, 2017[6] | Multiple | IPI (10 mg/kg/q3 wks) | IPI (3 mg/kg/q3 wks) | 14.5(I)/11.2(C) | 365 | 362 | 62 | 62 | 40 | 36 |
| Hamid, 2017[7] | Multiple | PEM (10 mg/kg/q3 wks) | DTIC + PTX, PTX, DTIC, or TEM | 28.1 | 181 | 179 | 60 | 63 | 40 | 36 |
| | | PEM (2 mg/kg/q3 wks) | | | 180 | 179 | 62 | 63 | 42 | 36 |
| Weber, 2017[8] | Multiple | NIV (3 mg/kg/q2 wks) | IPI (10 mg/kg/q3 wks) | 18 | 453 | 453 | 56 | 54 | 43 | 41 |
| Wolchok, 2017[9] | Multiple | NIV (3 mg/kg/q2 wks) | IPI (3 mg/kg/q3 wks) | 35.7(I)/18.6(C) | 313 | 311 | 59 | 61 | 36 | 36 |
| | | NIV (1 mg/kg/q3 wks) + IPI (3 mg/kg/q3 wks) | | 38(I)/18.6(C) | 313 | 311 | 59 | 61 | 34 | 36 |

**Abbreviations:** C: comparator; CBP: carboplatin; DTIC: dacarbazine; I: intervention; IPI: ipilimumab; MN: multination; NIV: nivolumab; NR: not reported; PEM: pembrolizumab; PTX: paclitaxel; TEM: temozolomide; wks: weeks.

©2020 Chang C-Y et al. *JAMA Network Open*

**eTable 3.** Frequencies of Any Immune-Related AEs in the Included Studies (n=9)

| Author, year/Treatment regimens | Wolchok, 2010[1] | Robert, 2014[2] | Robert, 2015[3] | Weber, 2015[4] | Hodi, 2017[5] | Ascierto, 2017[6] | Hamid, 2017[7] | Weber, 2017[8] | Wolchok, 2017[9] | Total |
|---|---|---|---|---|---|---|---|---|---|---|
| Threshold* (AE reporting rate) | 5% | 10% | NR | 1% | NR | NR | 5% | 10% | 5% | N/A |
| IPI (3 mg/kg/q3 wks) | 46 | N/A | 187 | N/A | 43 | 197 | N/A | N/A | 268 | 741 |
| IPI (10 mg/kg/q3 wks) | 50 | N/A | N/A | N/A | N/A | 269 | N/A | 434 | N/A | 753 |
| NIV (3 mg/kg/q2 wks) | N/A | 153 | N/A | 181 | N/A | N/A | N/A | 385 | 270 | 989 |
| NIV (1 mg/kg/q3 wks) + IPI (3 mg/kg/q3 wks) | N/A | N/A | N/A | N/A | 86 | N/A | N/A | N/A | 300 | 386 |
| PEM (2 mg/kg/q3 wks) | N/A | N/A | N/A | N/A | N/A | N/A | 125 | N/A | N/A | 125 |
| PEM (10 mg/kg/q2 wks) | N/A | N/A | 221 | N/A | N/A | N/A | N/A | N/A | N/A | 221 |
| PEM (10 mg/kg/q3 wks) | N/A | N/A | 202 | N/A | N/A | N/A | 136 | N/A | N/A | 338 |
| Chemotherapy[a] | N/A | 155 | N/A | 81 | N/A | N/A | 168 | N/A | N/A | 404 |

**Abbreviations**: **IPI**: ipilimumab; **NIV**: nivolumab; **PEM**: pembrolizumab. **N/A**: not applicable. **NR**: not reported.
*Threshold: The percentage of AEs the authors established as needing to reach to report AEs.
[a] Immune-related AEs are defined as AEs caused by ICIs, not chemotherapies (i.e., carboplatin, dacarbazine, and paclitaxel). For chemotherapy users, the AEs we identified are chemotherapy-related AEs.

©2020 Chang C-Y et al. *JAMA Network Open*

**eTable 4.** Probability Ranking of Being the Best Treatment Regimen With the Lowest Risk of Severe Immune-Related Adverse Events

| **Treatment regimens** | **Median rank**[b] | **95% CrI**[c] |
|---|---|---|
| NIV (3 mg/kg/q2 wks) | 1 | 1-5 |
| PEM (10 mg/kg/q3 wks) | 2 | 1-5 |
| PEM (2 mg/kg/q3 wks) | 3 | 1-7 |
| PEM (10 mg/kg/q2 wks) | 4 | 1-6 |
| IPI (3 mg/kg/q3 wks) | 5 | 2-6 |
| Chemotherapy[d] | 6 | 3-7 |
| IPI (10 mg/kg/q3 wks) | 7 | 5-8 |
| NIV (1 mg/kg/q3 wks)+ IPI (3 mg/kg/q3 wks) | 7 | 6-8 |

**Abbreviations: CrI**: credible interval; **IPI**: ipilimumab; **NIV**: nivolumab; **PEM**: pembrolizumab.
[a] We ranked the probability of being the best treatment regimen with the lowest risk of immune-related AEs by estimating the median and the 95% credible intervals of the posterior distribution for the rank of each treatment regimen. The lowest rank means the best treatment regimen (lowest risk of immune-related AEs).
[b] Median rank refers to median of the posterior distribution for the rank of each treatment.
[c] 95% CrI refers to 95% CrI of the posterior distribution for the rank of each treatment.
[d] Immune-related AEs are defined as AEs caused by ICIs, not chemotherapies (i.e., carboplatin, dacarbazine, and paclitaxel). For chemotherapy users, the AEs we identified are chemotherapy-related AEs.

**eTable 5.** Probability Ranking of Being the Best Treatment Regimen With the Lowest Risk of Individual Severe Immune-Related Adverse Events

| Treatment regimens | Median rank[b] | 95% CrI[c] | Median rank[b] | 95% CrI[c] | Median rank[b] | 95% CrI[c] |
|---|---|---|---|---|---|---|
| **Dermatologic AEs** | **Pruritus** | | **Rash** | | **Vitiligo** | |
| Chemotherapy[d] | 5 | 1-8 | 3 | 1-7 | 6 | 1-8 |
| IPI (3 mg/kg/q3 wks) | 5 | 2-7 | 7 | 4-8 | 4 | 1-8 |
| IPI (10 mg/kg/q3 wks) | 6 | 3-8 | 5 | 2-7 | 4 | 1-8 |
| PEM (10 mg/kg/q2 wks) | 2 | 1-7 | 3 | 1-8 | 4 | 1-8 |
| NIV (1 mg/kg/q3 wks)+ IPI (3 mg/kg/q3 wks) | 7 | 3-8 | 8 | 5-8 | 4 | 1-8 |
| NIV (3 mg/kg/q2 wks) | 3 | 1-6 | 3 | 1-6 | 5 | 1-8 |
| PEM (10 mg/kg/q3 wks) | 3 | 1-7 | 3 | 1-7 | 4 | 1-8 |
| PEM (2 mg/kg/q3 wks) | 4 | 1-8 | 2 | 1-8 | 5 | 1-8 |
| **Gastrointestinal AEs** | **Diarrhea** | | **Colitis** | | | |
| Chemotherapy[d] | 4 | 2-7 | 2 | 2-8 | | |
| IPI (3 mg/kg/q3 wks) | 6 | 3-7 | 6 | 4-8 | | |
| IPI (10 mg/kg/q3 wks) | 8 | 5-8 | 7 | 4-8 | | |
| PEM (10 mg/kg/q2 wks) | 5 | 1-8 | 4 | 2-7 | | |
| NIV (1 mg/kg/q3 wks)+ IPI (3 mg/kg/q3 wks) | 7 | 2-8 | 8 | 4-8 | | |
| NIV (3 mg/kg/q2 wks) | 3 | 1-6 | 3 | 2-6 | | |
| PEM (10 mg/kg/q3 wks) | 3 | 1-7 | 5 | 2-8 | | |
| PEM (2 mg/kg/q3 wks) | 1 | 1-5 | N/A | N/A | | |
| **Endocrine AEs** | **Hypothyroidism** | | **Hypophysis** | | | |
| Chemotherapy[d] | 5 | 1-8 | N/A | N/A | | |
| IPI (3 mg/kg/q3 wks) | 4 | 1-7 | 5 | 3-6 | | |
| IPI (10 mg/kg/q3 wks) | 5 | 1-8 | 6 | 2-6 | | |
| PEM (10 mg/kg/q2 wks) | 6 | 1-8 | 2 | 1-6 | | |
| NIV (1 mg/kg/q3 wks)+ IPI (3 mg/kg/q3 wks) | 4 | 1-8 | 4 | 1-6 | | |
| NIV (3 mg/kg/q2 wks) | 3 | 1-7 | 2 | 1-5 | | |
| PEM (10 mg/kg/q3 wks) | 4 | 1-8 | 2 | 1-6 | | |
| PEM (2 mg/kg/q3 wks) | 5 | 1-8 | N/A | N/A | | |

## eTable 5 (Continued).

| Treatment regimens | Median rank[b] | 95% CrI[c] | Median rank[b] | 95% CrI[c] | Median rank[b] | 95% CrI[c] |
|---|---|---|---|---|---|---|
| **Liver AEs** | **ALT increased** | | **AST increased** | | **Hepatitis** | |
| Chemotherapy[d] | 1 | 1-5 | 1 | 1-5 | N/A | N/A |
| IPI (3 mg/kg/q3 wks) | 2 | 1-3 | 2 | 1-3 | 1 | 1-3 |
| IPI (10 mg/kg/q3 wks) | 4 | 3-5 | 5 | 3-5 | 2 | 1-4 |
| PEM (10 mg/kg/q2 wks) | N/A | N/A | N/A | N/A | 3 | 1-4 |
| NIV (1 mg/kg/q3 wks)+ IPI (3 mg/kg/q3 wks) | 5 | 3-5 | 4 | 3-5 | N/A | N/A |
| NIV (3 mg/kg/q2 wks) | 2 | 1-4 | 2 | 1-4 | N/A | N/A |
| PEM (10 mg/kg/q3 wks) | N/A | N/A | N/A | N/A | 3 | 1-4 |
| PEM (2 mg/kg/q3 wks) | N/A | N/A | N/A | N/A | N/A | N/A |
| **Pulmonary AEs** | **Pneumonitis** | | | | | |
| Chemotherapy[d] | 5 | 1-7 | | | | |
| IPI (3 mg/kg/q3 wks) | 3 | 1-6 | | | | |
| IPI (10 mg/kg/q3 wks) | 7 | 2-7 | | | | |
| PEM (10 mg/kg/q2 wks) | 2 | 1-7 | | | | |
| NIV (1 mg/kg/q3 wks)+ IPI (3 mg/kg/q3 wks) | 5 | 1-7 | | | | |
| NIV (3 mg/kg/q2 wks) | 3 | 1-6 | | | | |
| PEM (10 mg/kg/q3 wks) | 3 | 1-7 | | | | |
| PEM (2 mg/kg/q3 wks) | N/A | N/A | | | | |

**Abbreviations: AEs**: adverse events; **ALT**: alanine aminotransferase; **AST**: aspartate aminotransferase; **CrI**: credible interval; **IPI**: ipilimumab; **NIV**: nivolumab; **N/A**: not applicable. No specific individual immune-related AE was reported in that treatment regimen in the included studies, so the indirect evidence cannot be generated. (For example, pneumonitis was not reported in any included studies comparing PEM (2 mg/kg/q3 wks) with other treatments, so we cannot generate the indirect evidence of pneumonitis from PEM (2 mg/kg/q3 wks) by linking that with other treatments); **PEM**: pembrolizumab.

[a] We ranked the probability of being the best treatment regimen with the lowest risk of immune-related AEs by estimating the median and the 95% credible intervals of the posterior distribution for the rank of each treatment regimen. The lowest rank means the best treatment regimen (lowest risk of immune-related AEs).

[b] Median rank refers to median of the posterior distribution for the rank of each treatment.

[c] 95% CrI refers to 95% CrI of the posterior distribution for the rank of each treatment[d] Immune-related AEs are defined as AEs caused by ICIs, not chemotherapies (i.e., carboplatin, dacarbazine, and paclitaxel). For chemotherapy users, the AEs we identified are chemotherapy-related AEs.

**eTable 6.** Secondary Outcomes of Individual Skin-Related Immune-Related Adverse Events by Severity

| Treatments | Comparators | Pruritus | | Rash | | Vitiligo | |
|---|---|---|---|---|---|---|---|
| | | OR (95%CrI) | | OR (95%CrI) | | OR (95%CrI) | |
| | | Grade 1-5 | Grade ≥3 | Grade 1-5 | Grade ≥3 | Grade 1-5 | Grade ≥3 |
| **PEM (2 mg/kg/q3 wks)** | Chemotherapy[a] | 6.78 (1.38-44.50)* | NR | 3.32 (0.64-16.57) | 0.78 (0.002-96.33) | NR | NR |
| | IPI (3 mg/kg/q3 wks) | 1.11 (0.13-9.81) | NR | 0.73 (0.10-5.30) | 0.08 (0.0001-11.94) | 3.09 (0.06-86.02) | NR |
| | IPI (10 mg/kg/q3 wks) | 1.03 (0.10-9.15) | 0.19 (0.0003-68.43) | 0.92 (0.11-7.42) | 0.19 (0.0002-31.94) | NR | NR |
| | PEM (10 mg/kg/q2 wks) | 1.05 (0.07-15.67) | NR | 0.73 (0.06-8.52) | NR | 0.56 (0.004-27.54) | NR |
| | NIV (1 mg/kg/q3 wks) + IPI (3 mg/kg/q3 wks) | 1.00 (0.08-11.61) | 0.1 (0.0001-47.11) | 0.46 (0.05-4.29) | 0.03 (0.00004-50.67) | NR | NR |
| | NIV (3 mg/kg/q2 wks) | 2.14 (0.28-16.47) | NR | 0.98 (0.14-6.26) | 0.56 (0.001-83.31) | 1.01 (0.02-20.78) | NR |
| | PEM (10 mg/kg/q3 wks) | 0.99 (0.12-8.07) | NR | 0.90 (0.13-6.22) | NR | 0.62 (0.02-13.01) | NR |
| | | | | | | | |
| **PEM (10 mg/kg/q3 wks)** | Chemotherapy[a] | 6.88 (1.90-32.58)* | 0.34 (0.003-13.45) | 3.70 (0.97-13.64) | 1.17 (0.02-49.24) | NR | 0.52 (0.003-51.62) |
| | IPI (3 mg/kg/q3 wks) | 1.12 (0.29-4.41) | 0.24 (0.01-4.9) | 0.81 (0.23-2.88) | 0.12 (0.002-2.36) | 5.04 (0.45-45.98) | NR |
| | IPI (10 mg/kg/q3 wks) | 1.04 (0.21-4.98) | 0.13 (0.002-4.24) | 1.03 (0.23-4.49) | 0.28 (0.004-8.62) | NR | NR |
| | PEM (10 mg/kg/q2 wks) | 1.06 (0.14-8.82) | NR | 0.81 (0.12-5.41) | NR | 0.92 (0.02-19.19) | NR |
| | NIV (1 mg/kg/q3 wks) + IPI (3 mg/kg/q3 wks) | 1.01 (0.16-5.91) | 0.06 (0.001-2.61) | 0.52 (0.09-2.67) | 0.05 (0.001-1.37) | 2.64 (0.07-73.98) | NR |
| | NIV (3 mg/kg/q2 wks) | 2.18 (0.49-9.61) | 1.06 (0.02-42.52) | 1.09 (0.27-4.21) | 0.82 (0.01-22.75) | 1.67 (0.09-17.85) | 0.58 (0.002-87.66) |

©2020 Chang C-Y et al. *JAMA Network Open*

**eTable 6 (Continued).**

| Treatments | Comparators | Pruritus | | Rash | | Vitiligo | |
|---|---|---|---|---|---|---|---|
| | | OR (95%Crl) | | OR (95%Crl) | | OR (95%Crl) | |
| | | Grade 1-5 | Grade ≥3 | Grade 1-5 | Grade ≥3 | Grade 1-5 | Grade ≥3 |
| **NIV (3 mg/kg/q2 wks)** | Chemotherapy[a] | 3.15 (1.17-11.29)* | 0.33 (0.01-4.68) | 3.41 (1.15-10.11)* | 1.53 (0.10-26.1) | NR | 0.84 (0.02-35.02) |
| | IPI (3 mg/kg/q3 wks) | 0.52 (0.17-1.58) | 0.23 (0.01-2.66) | 0.74 (0.27-2.15) | 0.14 (0.02-0.87) | 2.93 (0.39-33.23) | NR |
| | IPI (10 mg/kg/q3 wks) | 0.48 (0.14-1.49) | 0.12 (0.01-1.26) | 0.94 (0.32-2.85) | 0.35 (0.07-1.76) | 2.87 (0.12-98.34) | NR |
| | PEM (10 mg/kg/q2 wks) | 0.49 (0.07-3.64) | NR | 0.74 (0.12-4.79) | NR | 0.52 (0.02-16.02) | NR |
| | NIV (1 mg/kg/q3 wks) + IPI (3 mg/kg/q3 wks) | 0.47 (0.09-2.28) | 0.06 (0.002-1.18) | 0.47 (0.1-2.08) | 0.06 (0.004-0.49)* | 1.46 (0.07-53.11) | NR |
| | | | | | | | |
| **NIV (1 mg/kg/q3 wks) + IPI (3 mg/kg/q3 wks)** | Chemotherapy[a] | 6.73 (1.29-50.07)* | NR | 7.20 (1.31-38.56)* | NR | NR | NR |
| | IPI (3 mg/kg/q3 wks) | 1.11 (0.36-3.64) | 3.52 (0.42-43.73) | 1.56 (0.54-4.81) | 2.42 (0.57-15.03) | 1.95 (0.15-23.95) | NR |
| | IPI (10 mg/kg/q3 wks) | 1.03 (0.22-4.69) | 1.95 (0.10-37.14) | 1.97 (0.48-8.70) | 6.04 (0.74-72.79) | 1.94 (0.06-62.34) | NR |
| | PEM (10 mg/kg/q2 wks) | 1.05 (0.15-8.38) | NR | 1.55 (0.25-10.10) | NR | 0.34 (0.01-11.23) | NR |
| | | | | | | | |
| **PEM (10 mg/kg/q2 wks)** | Chemotherapy[a] | 6.44 (0.86-60.21) | 0.16 (0.0002-24.99) | 4.59 (0.63-32.49) | NR | NR | NR |
| | IPI (3 mg/kg/q3 wks) | 1.05 (0.20-5.19) | 0.13 (0.0003-4.46) | 1.01 (0.22-4.54) | 0.12(0.0002-4.01) | 5.77 (0.46-92.17) | NR |
| | IPI (10 mg/kg/q3 wks) | 0.98 (0.13-6.07) | 0.06 (0.0001-3.63) | 1.27 (0.21-7.44) | 0.28 (0.0004-15.26) | NR | NR |
| | | | | | | | |

**eTable 6 (Continued).**

| Treatments | Comparators | Pruritus | | Rash | | Vitiligo | |
|---|---|---|---|---|---|---|---|
| | | OR (95%CrI) | | OR (95%CrI) | | OR (95%CrI) | |
| | | Grade 1-5 | Grade ≥3 | Grade 1-5 | Grade ≥3 | Grade 1-5 | Grade ≥3 |
| **IPI (10 mg/kg/q3 wks)** | Chemotherapy[a] | 6.62 (1.73-36.71)* | 2.68 (0.07-92.85) | 3.65 (0.85-14.98) | 4.39 (0.20-97.3) | NR | NR |
| | IPI (3 mg/kg/q3 wks) | 1.08 (0.41-3.13) | 1.86 (0.29-14.38) | 0.79 (0.31-2.05) | 0.41 (0.10-2.04) | 1.01(0.08-11.72) | 1.09 (0.04-25.98) |
| | | | | | | | |
| **IPI (3 mg/kg/q3 wks)** | Chemotherapy[a] | 6.09 (1.70-29.16)* | 1.42 (0.04-46.21) | 4.59 (1.18-16.88)* | NR | 4.51 (0.34-61.74) | 0.38 (0.002-51.1) |

**Abbreviations: CrI**: credible interval; **IPI**: ipilimumab; **NIV**: nivolumab; **NR**: not reportable due to small sample sizes; N/A: not applicable. No specific individual immune-related AE was reported in that treatment regimen in the included studies, so the indirect evidence cannot be generated. (For example, pneumonitis was not reported in any included studies comparing PEM (2 mg/kg/q3 wks) with other treatments, so we cannot generate the indirect evidence of pneumonitis from PEM (2 mg/kg/q3 wks) by linking that with other treatments); OR: odds ratio; PEM: pembrolizumab.
*****indicates statistical significant.
[a] Immune-related AEs are defined as AEs caused by ICIs, not chemotherapies (i.e., carboplatin, dacarbazine, and paclitaxel). For chemotherapy users, the AEs we identified are chemotherapy-related AEs.

©2020 Chang C-Y et al. *JAMA Network Open*

**eTable 7.** Secondary Outcomes of Individual Gastrointestinal Tract-Related Immune-Related Adverse Events by Severity

| Treatments | Comparators | Diarrhea | | Colitis | |
|---|---|---|---|---|---|
| | | OR (95%Crl) | | OR (95%Crl) | |
| | | Grade 1-5 | Grade ≥3 | Grade 1-5 | Grade ≥3 |
| | | | | N/A | N/A |
| PEM (2 mg/kg/q3 wks) | Chemotherapy[a] | 1.14 (0.47-2.77) | 0.06 (0.0001-1.73) | | |
| | IPI (3 mg/kg/q3 wks) | 0.57 (0.2-1.64) | 0.03 (0.0001-1.33) | | |
| | IPI (10 mg/kg/q3 wks) | 0.46 (0.15-1.33) | 0.01 (0.00002-0.44)* | | |
| | PEM (10 mg/kg/q2 wks) | 0.80 (0.24-2.84) | 0.03 (0.0001-2.58) | | |
| | NIV (1 mg/kg/q3 wks) + IPI (3 mg/kg/q3 wks) | 0.35 (0.11-1.1) | 0.02 (0.00004-1.28) | | |
| | NIV (3 mg/kg/q2 wks) | 1.17 (0.43-3.25) | 0.07 (0.0001-2.98) | | |
| | PEM (10 mg/kg/q3 wks) | 0.89 (0.33-2.37) | 0.07 (0.0001-2.47) | | |
| | | | | | |
| PEM (10 mg/kg/q3 wks) | Chemotherapy[a] | 1.29 (0.66-2.53) | 0.89 (0.13-6.16) | NR | NR |
| | IPI (3 mg/kg/q3 wks) | 0.64 (0.35-1.19) | 0.43 (0.07-2.76) | 0.42 (0.05-3.3) | 0.33 (0.02-4.11) |
| | IPI (10 mg/kg/q3 wks) | 0.52 (0.25-1.04) | 0.12 (0.01-1.14) | 0.44(0.03-5.68) | 0.25 (0.01-5.85) |
| | PEM (10 mg/kg/q2 wks) | 0.90 (0.39-2.21) | 0.54 (0.04-7.55) | 2.21(0.11-42.8) | 1.86 (0.05-74.74) |
| | NIV (1 mg/kg/q3 wks) + IPI (3 mg/kg/q3 wks) | 0.39 (0.18-0.87)* | 0.31 (0.03-3.44) | 0.26 (0.02-2.73) | 0.17 (0.01-3.09) |
| | NIV (3 mg/kg/q2 wks) | 1.33 (0.70-2.62) | 1.02 (0.14-8.07) | 2.33 (0.12-44.96) | NR |
| | | | | | |
| NIV (3 mg/kg/q2 wks) | Chemotherapy[a] | 0.98 (0.57-1.60) | 0.85 (0.14-5.10) | NR | NR |
| | IPI (3 mg/kg/q3 wks) | 0.48 (0.30-0.78)* | 0.42 (0.10-1.72) | 0.18 (0.02-1.46) | 0.11 (0.01-1.64) |
| | IPI (10 mg/kg/q3 wks) | 0.39 (0.23-0.61)* | 0.12 (0.02-0.54)* | 0.19 (0.01-2.54) | 0.08 (0.003-2.12) |
| | PEM (10 mg/kg/q2 wks) | 0.68 (0.30-1.60) | 0.52 (0.04-6.01) | 0.95 (0.04-20.41) | 0.63 (0.02-29.38) |
| | NIV (1 mg/kg/q3 wks) + IPI (3 mg/kg/q3 wks) | 0.30 (0.15-0.58)* | 0.31 (0.04-2.40) | 0.11 (0.01-1.23) | 0.06 (0.002-1.13) |
| | | | | | |

©2020 Chang C-Y et al. *JAMA Network Open*

**eTable 7 (Continued).**

| Treatments | Comparators | Diarrhea | | Colitis | |
|---|---|---|---|---|---|
| | | OR (95%Crl) | | OR (95%Crl) | |
| | | Grade 1-5 | Grade ≥3 | Grade 1-5 | Grade ≥3 |
| **NIV (1 mg/kg/q3 wks) + IPI (3 mg/kg/q3 wks)** | Chemotherapy[a] | 3.27 (1.46-7.22)* | 2.77 (0.23-29.58) | NR | NR |
| | IPI (3 mg/kg/q3 wks) | 1.62 (0.98-2.78) | 1.36 (0.31-5.13) | 1.60 (0.42-8.95) | 1.92 (0.39-17.66) |
| | IPI (10 mg/kg/q3 wks) | 1.32 (0.65-2.52) | 0.40 (0.05-2.71) | 1.69 (0.23-17.86) | 1.46 (0.10-28.21) |
| | PEM (10 mg/kg/q2 wks) | 2.27 (0.97-5.59) | 1.69 (0.13-18.85) | NR | NR |
| | | | | | |
| **PEM (10 mg/kg/q2 wks)** | Chemotherapy[a] | 1.44 (0.55-3.51) | 1.68 (0.10-26.39) | NR | NR |
| | IPI (3 mg/kg/q3 wks) | 0.71 (0.35-1.42) | 0.81 (0.10-6.19) | 0.19 (0.02-1.66) | 0.18 (0.01-2.26) |
| | IPI (10 mg/kg/q3 wks) | 0.57 (0.24-1.27) | 0.23 (0.02-2.82) | 0.20 (0.01-2.79) | 0.13 (0.004-3.15) |
| | | | | | |
| **IPI (10 mg/kg/q3 wks)** | Chemotherapy[a] | 2.49 (1.29-4.94)* | 7.14 (0.79-67.23) | NR | NR |
| | IPI (3 mg/kg/q3 wks) | 1.23 (0.82-2.02) | 3.44 (0.83-15.2) | 0.95 (0.21-4.44) | 1.34 (0.19-11.72) |
| | | | | | |
| **IPI (3 mg/kg/q3 wks)** | Chemotherapy[a] | 2.02 (1.05-3.71)* | 2.06 (0.27-14.91) | NR | NR |

**Abbreviations: CrI**: credible interval; **IPI**: ipilimumab; **NIV**: nivolumab; **NR**: not reportable due to small sample sizes; **N/A**: not applicable. Nospecific individual immune-related AE was reported in that treatment regimen in the included studies, so the indirect evidence cannot be generated. (For example, pneumonitis was not reported in any included studies comparing PEM (2 mg/kg/q3 wks) with other treatments, so we cannot generate the indirect evidence of pneumonitis from PEM (2 mg/kg/q3 wks) by linking that with other treatments); OR: odds ratio; **PEM**: pembrolizumab.
*indicates statistical significant.
[a] Immune-related AEs are defined as AEs caused by ICIs, not chemotherapies (i.e., carboplatin, dacarbazine, and paclitaxel). For chemotherapy users, the AEs we identified are chemotherapy-related AEs.

©2020 Chang C-Y et al. *JAMA Network Open*

**eTable 8.** Secondary Outcomes of Individual Endocrine-Related Immune-Related Adverse Events by Severity

| Treatments | Comparators | Hypothyroidism | | Hypophysitis | |
|---|---|---|---|---|---|
| | | OR (95%Crl) | | OR (95%Crl) | |
| | | Grade 1-5 | Grade ≥3 | Grade 1-5 | Grade ≥3 |
| | | | | N/A | N/A |
| PEM (2 mg/kg/q3 wks) | Chemotherapy[a] | NR | NR | | |
| | IPI (3 mg/kg/q3 wks) | NR | NR | | |
| | IPI (10 mg/kg/q3 wks) | NR | NR | | |
| | PEM (10 mg/kg/q2 wks) | 0.93 (0.03-25.94) | NR | | |
| | NIV (1 mg/kg/q3 wks)+ IPI (3 mg/kg/q3 wks) | 1.87 (0.09-48.61) | NR | | |
| | NIV (3 mg/kg/q2 wks) | 2.54 (0.12-47.2) | NR | | |
| | PEM (10 mg/kg/q3 wks) | 1.09 (0.09-12.44) | NR | | |
| | | | | | |
| PEM (10 mg/kg/q3 wks) | Chemotherapy[a] | NR | 0.57 (0.003-57.87) | N/A | N/A |
| | IPI (3 mg/kg/q3 wks) | 5.01 (0.84-32.08) | NR | 0.26 (0.02-2.72) | 0.18 (0.002-3.71) |
| | IPI (10 mg/kg/q3 wks) | 3.99 (0.41-39.97) | NR | 0.25 (0.01-7.86) | 0.06 (0.0002-4.77) |
| | PEM (10 mg/kg/q2 wks) | 0.85 (0.08-9.24) | 0.44 (0.002-62.69) | NR | NR |
| | NIV (1 mg/kg/q3 wks)+ IPI (3 mg/kg/q3 wks) | 1.70 (0.21-17.91) | NR | 0.12 (0.01-1.86) | 0.21 (0.002-8.37) |
| | NIV (3 mg/kg/q2 wks) | 2.29 (0.26-18.72) | NR | 1.95 (0.07-53.66) | 0.69 (0.004-40.06) |
| | | | | | |
| NIV (3 mg/kg/q2 wks) | Chemotherapy[a] | NR | 0.35 (0.003-50.98) | N/A | N/A |
| | IPI (3 mg/kg/q3 wks) | 2.16 (0.58-9.98) | 0.83 (0.02-24.63) | 0.13 (0.01-0.91)* | 0.25 (0.02-4.61) |
| | IPI (10 mg/kg/q3 wks) | 1.73 (0.43-7.98) | 0.36 (0.01-6.76) | 0.13 (0.02-0.74)* | 0.10 (0.01-0.92)* |
| | PEM (10 mg/kg/q2 wks) | 0.36 (0.04-4.17) | 0.27 (0.001-80.28) | 1.16 (0.04-69.06) | NR |
| | NIV (1 mg/kg/q3 wks)+ IPI (3 mg/kg/q3 wks) | 0.71 (0.14-6.10) | 0.60 (0.01-39.46) | 0.06 (0.01-0.65)* | 0.31 (0.01-10.71) |
| | | | | | |

©2020 Chang C-Y et al. *JAMA Network Open*

eTable 8 (Continued).

| Treatments | Comparators | Hypothyroidism OR (95%Crl) | | Hypophysitis OR (95%Crl) | |
|---|---|---|---|---|---|
| | | **Grade 1-5** | **Grad ≥3** | Grade 1-5 | **Grade ≥3** |
| **NIV (1 mg/kg/q3 wks)+ IPI (3 mg/kg/q3 wks)** | Chemotherapy[a] | NR | NR | N/A | N/A |
| | IPI (3 mg/kg/q3 wks) | 2.98 (0.71-9.84) | 1.27 (0.05-46.99) | 2.09 (0.50-9.43) | 0.80 (0.11-5.87) |
| | IPI (10 mg/kg/q3 wks) | 2.43 (0.28-15.18) | 0.60 (0.01-49.95) | 2.06 (0.16-32.96) | 0.32 (0.003-9.87) |
| | PEM (10 mg/kg/q2 wks) | 0.49 (0.04-4.39) | NR | NR | NR |
| | | | | | |
| **PEM (10 mg/kg/q2 wks)** | Chemotherapy[a] | NR | NR | N/A | N/A |
| | IPI (3 mg/kg/q3 wks) | 5.98 (0.92-40.69) | NR | 0.11 (0.003-1.43) | 0.17 (0.003-3.46) |
| | IPI (10 mg/kg/q3 wks) | 4.79 (0.41-51.00) | NR | 0.11 (0.002-3.57) | 0.06 (0.0002-4.47) |
| | | | | | |
| **IPI (10 mg/kg/q3 wks)** | Chemotherapy[a] | NR | NR | N/A | N/A |
| | IPI (3 mg/kg/q3 wks) | 1.24 (0.28-5.94) | 2.19 (0.07-91.41) | 1.02 (0.10-9.37) | NR |
| | | | | | |
| **IPI (3 mg/kg/q3 wks)** | Chemotherapy[a] | NR | 0.44 (0.003-73.97) | N/A | N/A |

**Abbreviations: CrI**: credible interval; **IPI**: ipilimumab; **NIV**: nivolumab; **NR**: not reportable due to small sample sizes; **N/A**: not applicable. No specific individual immune-related AE was reported in that treatment regimen in the included studies, so the indirect evidence cannot be generated. (For example, pneumonitis was not reported in any included studies comparing PEM (2 mg/kg/q3 wks) with other treatments, so we cannot generate the indirect evidence of pneumonitis from PEM (2 mg/kg/q3 wks) by linking that with other treatments); OR: odds ratio; **PEM**: pembrolizumab.
***indicates statistical significant.
[a] Immune-related AEs are defined as AEs caused by ICIs, not chemotherapies (i.e., carboplatin, dacarbazine, and paclitaxel). For chemotherapy users, the AEs we identified are chemotherapy-related AEs.

©2020 Chang C-Y et al. *JAMA Network Open*

**eTable 9.** Secondary Outcomes of Individual Liver-Related Immune-Related Adverse Events by Severity

| **Treatments** | **Comparators** | **ALT increased** | | **AST increased** | | **Hepatitis** | |
|---|---|---|---|---|---|---|---|
| | | **OR (95%CrI)** | | **OR (95%CrI)** | | **OR (95%CrI)** | |
| | | **Grade 1-5** | **Grade ≥3** | **Grade 1-5** | **Grade ≥3** | **Grade 1-5** | **Grade ≥3** |
| | | **N/A** | **N/A** | | | **N/A** | **N/A** |
| **PEM (2 mg/kg/q3 wks)** | Chemotherapy[a] | | | | | | |
| | IPI (3 mg/kg/q3 wks) | | | | | | |
| | IPI (10 mg/kg/q3 wks) | | | | | | |
| | PEM (10 mg/kg/q2 wks) | | | | | | |
| | NIV (1 mg/kg/q3 wks) + IPI (3 mg/kg/q3 wks) | | | | | | |
| | NIV (3 mg/kg/q2 wks) | | | | | | |
| | PEM (10 mg/kg/q3 wks) | | | | | | |
| | | **N/A** | **N/A** | | | | |
| **PEM (10 mg/kg/q3 wks)** | Chemotherapy[a] | | | | | N/A | N/A |
| | IPI (3 mg/kg/q3 wks) | | | | | 1.68 (0.09-30.1) | NR |
| | IPI (10 mg/kg/q3 wks) | | | | | 0.60 (0.01-37.56) | NR |
| | PEM (10 mg/kg/q2 wks) | | | | | 1.81 (0.04-84.64) | 1.83(0.04-83.96) |
| | NIV (1 mg/kg/q3 wks) + IPI (3 mg/kg/q3 wks) | | | | | N/A | N/A |
| | NIV (3 mg/kg/q2 wks) | | | | | N/A | N/A |
| | | | | | | | |

©2020 Chang C-Y et al. *JAMA Network Open*

**eTable 9 (Continued).**

| Treatments | Comparators | ALT increased | | AST increased | | Hepatitis | |
|---|---|---|---|---|---|---|---|
| | | OR (95%Crl) | | OR (95%Crl) | | OR (95%Crl) | |
| | | Grade 1-5 | Grade ≥3 | Grade 1-5 | Grade ≥3 | Grade 1-5 | Grade ≥3 |
| | | | | | | **N/A** | **N/A** |
| **NIV (3 mg/kg/q2 wks)** | Chemotherapy[a] | NR | NR | NR | NR | | |
| | IPI (3 mg/kg/q3 wks) | 1.47 (0.30-8.06) | 0.91 (0.15-5.63) | 1.59 (0.34-8.11) | 1.28 (0.14-12.71) | | |
| | IPI (10 mg/kg/q3 wks) | 0.31 (0.05-1.40) | 0.16 (0.03-0.86)* | 0.30 (0.06-1.25) | 0.10 (0.01-0.76)* | | |
| | PEM (10 mg/kg/q2 wks) | N/A | N/A | N/A | N/A | | |
| | NIV (1 mg/kg/q3 wks) + IPI (3 mg/kg/q3 wks) | 0.26 (0.04-2.67) | 0.12 (0.01-1.12) | 0.31(0.05-2.61) | 0.12(0.01-1.69) | | |
| | | | | | | **N/A** | **N/A** |
| **NIV (1 mg/kg/q3 wks) + IPI (3 mg/kg/q3 wks)** | Chemotherapy[a] | NR | NR | NR | NR | | |
| | IPI (3 mg/kg/q3 wks) | 5.62 (1.19-21.46)* | 7.15 (1.57-47.37)* | 5.12(1.21-19.11)* | 10.42(1.72-92.95)* | | |
| | IPI (10 mg/kg/q3 wks) | 1.19 (0.1-8.42) | 1.27 (0.12-15.45) | 0.98(0.09-6.54) | 0.78(0.04-14.62) | | |
| | PEM (10 mg/kg/q2 wks) | N/A | N/A | N/A | N/A | | |
| | | **N/A** | **N/A** | **N/A** | **N/A** | | |
| **PEM (10 mg/kg/q2 wks)** | Chemotherapy[a] | | | | | N/A | N/A |
| | IPI (3 mg/kg/q3 wks) | | | | | 0.92 (0.10-18.16) | NR |
| | IPI (10 mg/kg/q3 wks) | | | | | 0.32 (0.01-22.14) | NR |

©2020 Chang C-Y et al. *JAMA Network Open*

**eTable 9 (Continued).**

| Treatments | Comparators | ALT increased OR (95%CrI) | | AST increased OR (95%CrI) | | Hepatitis OR (95%CrI) | |
|---|---|---|---|---|---|---|---|
| | | Grade 1-5 | Grade ≥3 | Grade 1-5 | Grade ≥3 | Grade 1-5 | Grade ≥3 |
| **IPI (10 mg/kg/q3 wks)** | Chemotherapy[a] | NR | NR | NR | NR | N/A | N/A |
| | IPI (3 mg/kg/q3 wks) | 4.79 (1.02-28.17)* | 5.75 (0.96-38.82) | 5.28(1.21-33.28)* | NR | 2.78 (0.15-60.73) | 2.76 (0.15-61.00) |
| | | | | | | | |
| **IPI (3 mg/kg/q3 wks)** | Chemotherapy[a] | NR | NR | NR | NR | N/A | N/A |

**Abbreviations: ALT**: alanine aminotransferase; **AST**: aspartate aminotransferase; **CrI**: credible interval; **IPI**: ipilimumab; **NIV**: nivolumab; **NR**: not reportable due to small sample sizes; **N/A**: not applicable. No specific individual immune-related AE was reported in that treatment regimen in the included studies, so the indirect evidence cannot be generated. (For example, pneumonitis was not reported in any included studies comparing PEM (2 mg/kg/q3 wks) with other treatments, so we cannot generate the indirect evidence of pneumonitis from PEM (2 mg/kg/q3 wks) by linking that with other treatments); **OR**: odds ratio; **PEM**: pembrolizumab.
*indicates statistical significant.
[a] Immune-related AEs are defined as AEs caused by ICIs, not chemotherapies (i.e., carboplatin, dacarbazine, and paclitaxel). For chemotherapy users, the AEs we identified are chemotherapy-related AEs.

**eTable 10.** Secondary Outcomes of Individual Pulmonary-Related Immune-Related Adverse Events by Severity

| **Treatments** | **Comparators** | **Pneumonitis** | |
|---|---|---|---|
| | | **OR (95%CrI)** | |
| | | **Grade 1-5** | **Grade ≥3** |
| | | **N/A** | **N/A** |
| **PEM (2 mg/kg/q3 wks)** | Chemotherapy[a] | | |
| | IPI (3 mg/kg/q3 wks) | | |
| | IPI (10 mg/kg/q3 wks) | | |
| | PEM (10 mg/kg/q2 wks) | | |
| | NIV (1 mg/kg/q3 wks)+ IPI (3 mg/kg/q3 wks) | | |
| | NIV (3 mg/kg/q2 wks) | | |
| | PEM (10 mg/kg/q3 wks) | | |
| | | | |
| **PEM (10 mg/kg/q3 wks)** | Chemotherapy[a] | NR | NR |
| | IPI (3 mg/kg/q3 wks) | NR | NR |
| | IPI (10 mg/kg/q3 wks) | NR | NR |
| | PEM (10 mg/kg/q2 wks) | NR | NR |
| | NIV (1 mg/kg/q3 wks) + IPI (3 mg/kg/q3 wks) | NR | NR |
| | NIV (3 mg/kg/q2 wks) | NR | NR |
| | | | |
| **NIV (3 mg/kg/q2 wks)** | Chemotherapy[a] | NR | NR |
| | IPI (3 mg/kg/q3 wks) | 1.01 (0.07-15.17) | 0.96 (0.01-56.66) |
| | IPI (10 mg/kg/q3 wks) | 0.05 (0.00004-5.37) | 0.05 (0.00004-14.13) |
| | PEM (10 mg/kg/q2 wks) | NR | NR |
| | NIV (1 mg/kg/q3 wks) + IPI (3 mg/kg/q3 wks) | 0.20 (0.01-7.31) | 0.24 (0.002-14.00) |
| | | | |

©2020 Chang C-Y et al. *JAMA Network Open*

**eTable 10 (Continued).**

| Treatments | Comparators | Pneumonitis | |
|---|---|---|---|
| | | OR (95%Crl) | |
| | | Grade 1-5 | Grade ≥3 |
| **NIV (1 mg/kg/q3 wks) + IPI (3 mg/kg/q3 wks)** | Chemotherapy[a] | NR | NR |
| | IPI (3 mg/kg/q3 wks) | NR | NR |
| | IPI (10 mg/kg/q3 wks) | NR | NR |
| | PEM (10 mg/kg/q2 wks) | NR | NR |
| | | | |
| **PEM (10 mg/kg/q2 wks)** | Chemotherapy[a] | NR | NR |
| | IPI (3 mg/kg/q3 wks) | 0.93 (0.02-62.37) | 0.29 (0.0003-32.25) |
| | IPI (10 mg/kg/q3 wks) | 0.04 (0.00002-13.1) | 0.01 (0.000003-7.07) |
| | | | |
| **IPI (10 mg/kg/q3 wks)** | Chemotherapy[a] | NR | NR |
| | IPI (3 mg/kg/q3 wks) | NR | NR |
| | | | |
| | | | |
| **IPI (3 mg/kg/q3 wks)** | Chemotherapy[a] | NR | NR |

**Abbreviations: CrI**: credible interval; **IPI**: ipilimumab; **NIV**: nivolumab; **NR**: not reportable due to small sample sizes; **N/A**: not applicable. No specific individual immune-related AE was reported in that treatment regimen in the included studies, so the indirect evidence cannot be generated. (For example, pneumonitis was not reported in any included studies comparing PEM (2 mg/kg/q3 wks) with other treatments, so we cannot generate the indirect evidence of pneumonitis from PEM (2 mg/kg/q3 wks) by linking that with other treatments); **OR**: odds ratio; **PEM**: pembrolizumab.
*indicates statistical significant.
[a] Immune-related AEs are defined as AEs caused by ICIs, not chemotherapies (i.e., carboplatin, dacarbazine, and paclitaxel). For chemotherapy users, the AEs we identified are chemotherapy-related AEs.

©2020 Chang C-Y et al. *JAMA Network Open*

**eTable 11.** Node-Splitting Analysis[a] of the Primary Outcome: Any Immune-Related Adverse Events

| Treatments | Comparators | Direct effect | | Indirect effect | | Inconsistency estimate | |
|---|---|---|---|---|---|---|---|
| | | **OR** | **95% CrI** | **OR** | **95% CrI** | **Ratio of the OR[b]** | **P-value[c]** |
| **PEM (2 mg/kg/q3 wks)** | Chemotherapy[d] | 0.58 | 0.15 to 2.17 | 1.14 | NR | 0.51 | 1.00 |
| | IPI (3 mg/kg/q3 wks) | N/A | N/A | 0.63 | 0.15 to 2.70 | N/A | 1.01 |
| | IPI (10 mg/kg/q3 wks) | N/A | N/A | 0.29 | 0.06 to 1.39 | N/A | 1.02 |
| | PEM (10 mg/kg/q2 wks) | N/A | N/A | 0.46 | 0.09 to 2.42 | N/A | 1.01 |
| | NIV (1 mg/kg/q3 wks) + IPI (3 mg/kg/q3 wks) | N/A | N/A | 0.29 | 0.05 to 1.56 | N/A | 1.02 |
| | NIV (3 mg/kg/q2 wks) | N/A | N/A | 0.89 | 0.22 to 3.62 | 1.23 | 1.01 |
| | PEM (10 mg/kg/q3 wks) | 0.73 | 0.20 to 2.69 | 1.93 | NR | 0.38 | 0.99 |
| | | | | | | | |
| **PEM (10 mg/kg/q3 wks)** | Chemotherapy[d] | 0.75 | 0.12 to 4.84 | 1.08 | 0.08 to 15.05 | 0.70 | 0.73 |
| | IPI (3 mg/kg/q3 wks) | 0.99 | 0.17 to 5.81 | 0.69 | 0.05 to 9.15 | 1.44 | 1.28 |
| | IPI (10 mg/kg/q3 wks) | N/A | N/A | 0.41 | 0.12 to 1.44 | N/A | 1.01 |
| | PEM (10 mg/kg/q2 wks) | 0.69 | 0.10 to 4.66 | 0.48 | 0.02 to 14.12 | 1.45 | 1.26 |
| | NIV (1 mg/kg/q3 wks) + IPI (3 mg/kg/q3 wks) | N/A | N/A | 0.41 | 0.10 to 1.65 | N/A | 1.01 |
| | NIV (3 mg/kg/q2 wks) | N/A | N/A | 1.26 | 0.41 to 3.90 | N/A | 1.00 |

©2020 Chang C-Y et al. *JAMA Network Open*

**eTable 11 (Continued).**

| Treatments | Comparators | Direct effect | | Indirect effect | | Inconsistency estimate | |
|---|---|---|---|---|---|---|---|
| | | OR | 95% CrI | OR | 95% CrI | Ratio of the OR[b] | P-value[C] |
| **NIV (3 mg/kg/q2 wks)** | Chemotherapy[d] | 0.71 | 0.18 to 2.77 | 0.48 | 0.02 to 10.03 | 1.47 | 1.28 |
| | IPI (3 mg/kg/q3 wks) | 1.04 | 0.25 to 4.22 | 0.51 | 0.15 to 1.67 | 2.05 | 1.72 |
| | IPI (10 mg/kg/q3 wks) | 0.25 | 0.04 to 1.37 | 0.44 | 0.07 to 2.87 | 0.56 | 0.44 |
| | PEM (10 mg/kg/q2 wks) | N/A | N/A | 0.52 | 0.13 to 2.12 | N/A | 1.01 |
| | NIV (1 mg/kg/q3 wks) + IPI (3 mg/kg/q3 wks) | 0.30 | 0.05 to 1.92 | 0.27 | 0.03 to 2.50 | 1.10 | 1.15 |
| **NIV (1 mg/kg/q3 wks) + IPI (3 mg/kg/q3 wks)** | Chemotherapy[d] | N/A | N/A | 2.07 | 0.54 to 7.89 | N/A | 1.00 |
| | IPI (3 mg/kg/q3 wks) | 2.26 | 0.76 to 6.74 | 2.25 | NR | 1.01 | 1.00 |
| | IPI (10 mg/kg/q3 wks) | N/A | N/A | 1.00 | 0.30 to 3.42 | N/A | 1.01 |
| | PEM (10 mg/kg/q2 wks) | N/A | N/A | 1.60 | 0.34 to 7.61 | N/A | 1.00 |
| **PEM (10 mg/kg/q2 wks)** | Chemotherapy[d] | N/A | N/A | 1.29 | 0.31 to 5.44 | N/A | 1.01 |
| | IPI (3 mg/kg/q3 wks) | 1.43 | 0.21 to 9.92 | 1.01 | 0.03 to 30.30 | 1.42 | 1.24 |
| | IPI (10 mg/kg/q3 wks) | N/A | N/A | 0.63 | 0.15 to 2.62 | 1.73 | 1.01 |
| **IPI (10 mg/kg/q3 wks)** | Chemotherapy[d] | N/A | N/A | 2.06 | 0.63 to 6.80 | N/A | 1.00 |
| | IPI (3 mg/kg/q3 wks) | 1.89 | 0.57 to 6.30 | 3.41 | 0.38 to 30.61 | 0.55 | 0.45 |
| **IPI (3 mg/kg/q3 wks)** | Chemotherapy[d] | N/A | N/A | 0.95 | 0.32 to 2.80 | N/A | 1.01 |

**Abbreviations: CrI**: credible interval; **IPI**: ipilimumab; **N/A**: not applicable. Nospecific individual immune-related AE was reported in that treatment regimen in the included studies, so the indirect evidence cannot be generated. (For example, pneumonitis was not reported in any included studies comparing PEM (2 mg/kg/q3 wks) with other treatments, so we cannot generate the indirect evidence of pneumonitis from PEM (2 mg/kg/q3 wks) by linking that with other treatments); **NIV**: nivolumab; **NR**: not reportable due to small sample sizes; OR: odds ratio; **PEM**: pembrolizumab; **SD**: standard deviations. [a] We compared posterior means and standard deviations of the log-odds ratios obtained from direct and indirect evidence for each treatment comparison, and then compared

©2020 Chang C-Y et al. *JAMA Network Open*

the inconsistency as direct effect minus indirect effect at each treatment comparison. [b] The ratio of the OR indicates ORdirect divied by ORindirect [c] P ≤ 0·05 indicates a significant inconsistency between the direct effect and indirect effects.[d] Immune-related AEs are defined as AEs caused by ICIs, not chemotherapies (i.e., carboplatin, dacarbazine, and paclitaxel). For chemotherapy users, the AEs we identified are chemotherapy-related AEs.

**eTable 12.** Node-Splitting Analysis[a] of the Primary Outcome: Severe Immune-Related Adverse Events

| Treatments | Comparators | Direct effect | | Indirect effect | | Inconsistency estimate | |
|---|---|---|---|---|---|---|---|
| | | **OR** | **95% CrI** | **OR** | **95% CrI** | **Ratio of the OR[b]** | **P-value[c]** |
| **PEM (2 mg/kg/q3 wks)** | Chemotherapy[d] | 0.42 | 0.08 to 2.27 | 0.99 | NR | 0.43 | 0.99 |
| | IPI (3 mg/kg/q3 wks) | N/A | N/A | 0.47 | 0.07 to 3.08 | N/A | N/A |
| | IPI (10 mg/kg/q3 wks) | N/A | N/A | 0.15 | 0.02 to 1.17 | N/A | N/A |
| | PEM (10 mg/kg/q2 wks) | N/A | N/A | 0.69 | 0.08 to 6.11 | N/A | N/A |
| | NIV (1 mg/kg/q3 wks)+ IPI (3 mg/kg/q3 wks) | N/A | N/A | 0.12 | 0.01 to 0.95 | N/A | N/A |
| | NIV (3 mg/kg/q2 wks) | N/A | N/A | 0.75 | 0.12 to 4.59 | N/A | N/A |
| | PEM (10 mg/kg/q3 wks) | 0.79 | 0.14 to 4.40 | 3.34 | NR | 0.24 | 1.00 |
| **PEM (10 mg/kg/q3 wks)** | Chemotherapy[d] | 0.56 | 0.06 to 5.13 | 0.30 | 0.01 to 7.11 | 0.62 | 1.36 |
| | IPI (3 mg/kg/q3 wks) | 0.45 | 0.05 to 3.98 | 0.85 | 0.04 to 19.54 | 1.90 | 0.63 |
| | IPI (10 mg/kg/q3 wks) | N/A | N/A | 0.18 | 0.04 to 0.89 | N/A | 1.01 |
| | PEM (10 mg/kg/q2 wks) | 0.72 | 0.08 to 6.40 | 1.34 | 0.03 to 59.88 | 0.54 | 0.68 |
| | NIV (1 mg/kg/q3 wks)+ IPI (3 mg/kg/q3 wks) | N/A | N/A | 0.14 | 0.02 to 0.73 | N/A | 1.01 |
| | NIV (3 mg/kg/q2 wks) | N/A | N/A | 0.87 | 0.21 to 3.71 | N/A | 1.00 |
| **NIV (3 mg/kg/q2 wks)** | Chemotherapy[d] | 0.47 | 0.10 to 2.25 | 0.90 | 0.03 to 28.23 | 0.52 | 0.63 |
| | IPI (3 mg/kg/q3 wks) | 0.71 | 0.07 to 6.72 | 0.62 | 0.10 to 3.82 | 1.14 | 1.12 |
| | IPI (10 mg/kg/q3 wks) | 5.08 | 0.50 to 52.07 | 0.21 | 0.02 to 2.65 | -0.05 | 0.94 |
| | PEM (10 mg/kg/q2 wks) | N/A | N/A | 0.93 | 0.15 to 5.63 | N/A | 1.00 |
| | NIV (1 mg/kg/q3 wks)+ IPI (3 mg/kg/q3 wks) | 0.19 | 0.02 to 1.71 | 0.12 | 0.01 to 1.73 | 1.60 | 1.34 |

©2020 Chang C-Y et al. *JAMA Network Open*

**eTable 12 (Continued).**

| Treatments | Comparators | Direct effect | | Indirect effect | | Inconsistency estimate | |
|---|---|---|---|---|---|---|---|
| | | OR | 95% CrI | OR | 95% CrI | Ratio of the OR[b] | P-value[c] |
| | | | | | | | |
| **NIV (1 mg/kg/q3 wks)+ IPI (3 mg/kg/q3 wks)** | Chemotherapy[d] | N/A | N/A | 3.38 | 0.67 to 16.96 | 0.26 | 0.99 |
| | IPI (3 mg/kg/q3 wks) | 4.28 | 1.27 to 14.42 | 0.70 | NR | 6.15 | 1.01 |
| | IPI (10 mg/kg/q3 wks) | N/A | N/A | 1.32 | 0.31 to 5.70 | N/A | 1.00 |
| | PEM (10 mg/kg/q2 wks) | N/A | N/A | 5.98 | 0.89 to 40.34 | N/A | 0.98 |
| | | | | | | | |
| **PEM (10 mg/kg/q2 wks)** | Chemotherapy[d] | N/A | N/A | 0.56 | 0.09 to 3.65 | 1.56 | 1.00 |
| | IPI (3 mg/kg/q3 wks) | 0.62 | 0.07 to 5.69 | 1.15 | 0.02 to 56.02 | -0.62 | 0.68 |
| | IPI (10 mg/kg/q3 wks) | N/A | N/A | 0.22 | 0.03 to 1.40 | N/A | 1.01 |
| | | | | | | | |
| **IPI (10 mg/kg/q3 wks)** | Chemotherapy[d] | N/A | N/A | 0.94 | 0.77 | N/A | 1.00 |
| | IPI (3 mg/kg/q3 wks) | 3.09 | 0.59 to 16.30 | 3.25 | 0.18 to 59.66 | 0.95 | 0.94 |
| | | | | | | | |
| **IPI (3 mg/kg/q3 wks)** | Chemotherapy[d] | N/A | N/A | 2.56 | 0.56 to 11.59 | N/A | 1.00 |

**Abbreviations: CrI**: credible interval; **IPI**: ipilimumab; **N/A**: not applicable. Nospecific individual immune-related AE was reported in that treatment regimen in the included studies, so the indirect evidence cannot be generated. (For example, pneumonitis was not reported in any included studies comparing PEM (2 mg/kg/q3 wks) with other treatments, so we cannot generate the indirect evidence of pneumonitis from PEM (2 mg/kg/q3 wks) by linking that with other treatments); **NIV**: nivolumab; **NR**: not reportable due to small sample sizes; OR: odds ratio; **PEM**: pembrolizumab; **SD**: standard deviations.

[a] We compared posterior means and standard deviations of the log-odds ratios obtained from direct and indirect evidence for each treatment comparison, and then compared the inconsistency as direct effect minus indirect effect at each treatment comparison. **[b]** The ratio of the OR indicates ORdirect divied by ORindirect. [c] P ≤ 0·05 indicates a significant inconsistency between the direct effect and indirect effects. **[d]** Immune-related AEs are defined as AEs caused by ICIs, not chemotherapies (i.e., carboplatin, dacarbazine, and paclitaxel). For chemotherapy users, the AEs we identified are chemotherapy-related AEs.

©2020 Chang C-Y et al. *JAMA Network Open*

**eFigure 1.** Preferred Reporting Items for Systematic Reviews and Meta-Analyses (PRISMA) Flow Diagram: Literature Search, Study Identification, Selection, and Exclusion

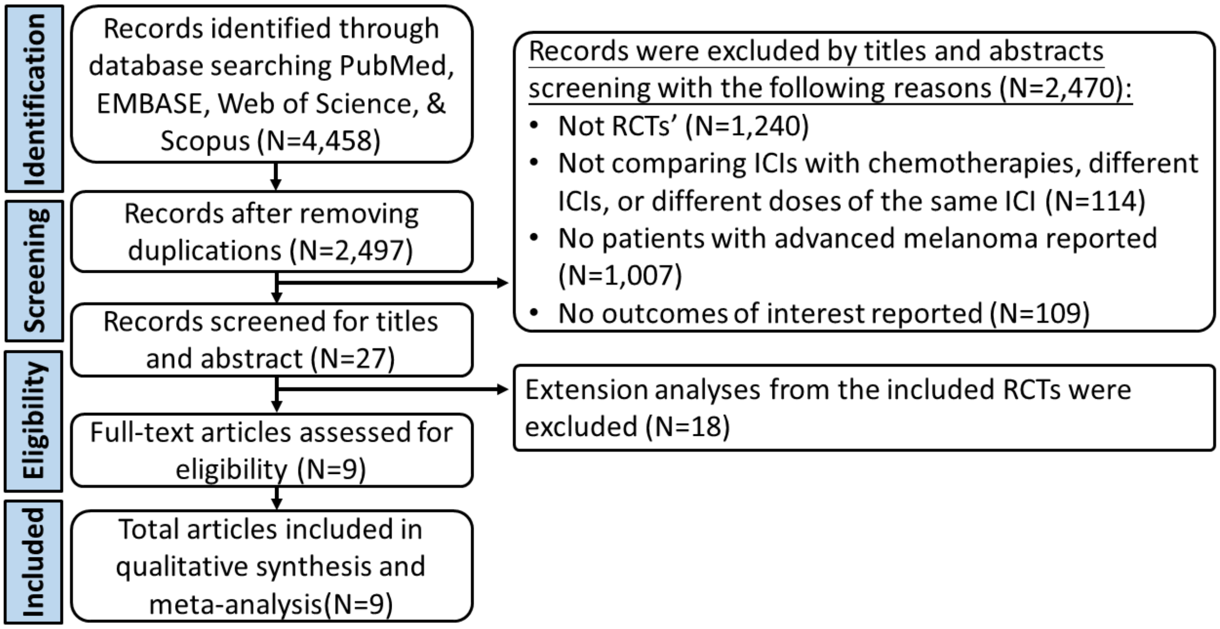


**Abbreviations:** ICIs: immune checkpoint inhibitors; RCT: randomized controlled trial.

**eFigure 2.** Risk of Bias Assessment for the 9 Included Randomized Controlled Trials

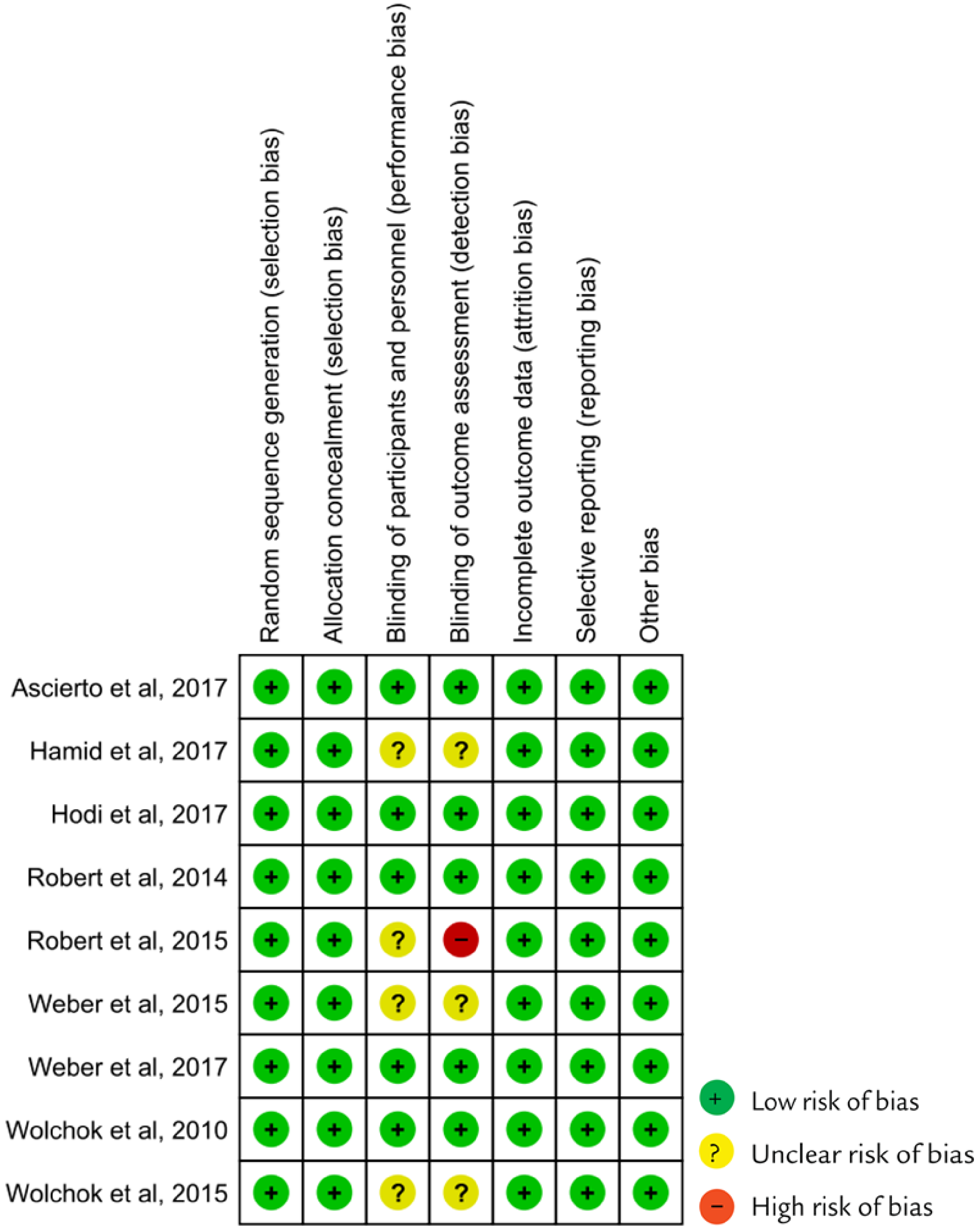


©2020 Chang C-Y et al. *JAMA Network Open*

## eReferences


1. Wolchok JD, Neyns B, Linette G, et al. Ipilimumab monotherapy in patients with pretreated advanced melanoma: a randomised, double-blind, multicentre, phase 2, dose-ranging study. *The Lancet Oncology.* 2010;11(2):155-164.
2. Robert C, Long GV, Brady B, et al. Nivolumab in previously untreated melanoma without BRAF mutation. *The New England journal of medicine.* 2015;372(4):320-330.
3. Robert C, Schachter J, Long GV, et al. Pembrolizumab versus Ipilimumab in Advanced Melanoma. *The New England journal of medicine.* 2015;372(26):2521-2532.
4. Weber JS, D'Angelo SP, Minor D, et al. Nivolumab versus chemotherapy in patients with advanced melanoma who progressed after anti-CTLA-4 treatment (CheckMate 037): a randomised, controlled, open-label, phase 3 trial. *The Lancet Oncology.* 2015;16(4):375-384.
5. Hodi FS, Chesney J, Pavlick AC, et al. Combined nivolumab and ipilimumab versus ipilimumab alone in patients with advanced melanoma: 2-year overall survival outcomes in a multicentre, randomised, controlled, phase 2 trial. *The Lancet Oncology.* 2016;17(11):1558-1568.
6. Ascierto PA, Del Vecchio M, Robert C, et al. Ipilimumab 10 mg/kg versus ipilimumab 3 mg/kg in patients with unresectable or metastatic melanoma: a randomised, double-blind, multicentre, phase 3 trial. *The Lancet Oncology.* 2017;18(5):611-622.
7. Hamid O, Puzanov I, Dummer R, et al. Final analysis of a randomised trial comparing pembrolizumab versus investigator-choice chemotherapy for ipilimumab-refractory advanced melanoma. *European journal of cancer (Oxford, England : 1990).* 2017;86:37-45.
8. Weber J, Mandala M, Del Vecchio M, et al. Adjuvant Nivolumab versus Ipilimumab in Resected Stage III or IV Melanoma. *The New England journal of medicine.* 2017;377(19):1824-1835.
9. Wolchok JD, Chiarion-Sileni V, Gonzalez R, et al. Overall Survival with Combined Nivolumab and Ipilimumab in Advanced Melanoma. *The New England journal of medicine.* 2017;377(14):1345-1356.


©2020 Chang C-Y et al. *JAMA Network Open*
